# Major Depressive Disorder associated dysregulation of ZBTB7A in orbitofrontal cortex promotes astrocyte-mediated stress susceptibility

**DOI:** 10.1016/j.neuron.2025.05.023

**Published:** 2025-06-13

**Authors:** Sasha L. Fulton, Jaroslav Bendl, Giuseppina Di Salvo, John F. Fullard, Amni Al-Kachak, Ashley E. Lepack, Andrew F. Stewart, Sumnima Singh, Wolfram C. Poller, Ryan M. Bastle, Mads E. Hauberg, Amanda K. Fakira, Vishwendra Patel, Min Chen, Romain Durand-de Cuttoli, Isabel Gameiro-Ros, Flurin Cathomas, Aarthi Ramakrishnan, Kelly Gleason, Li Shen, Carol A. Tamminga, Ana Milosevic, Scott J. Russo, Filip K. Swirski, Paul A. Slesinger, Ishmail Abdus-Saboor, Robert D. Blitzer, Panos Roussos, Ian Maze

**Affiliations:** 1 Friedman Brain Institute, Icahn School of Medicine at Mount Sinai, NY, NY USA; 2 Nash Family Department of Neuroscience, Icahn School of Medicine at Mount Sinai, NY, NY USA; 3 Department of Pharmacological Sciences, Icahn School of Medicine at Mount Sinai, NY, NY USA; 4 Center for Disease Neurogenomics, Icahn School of Medicine at Mount Sinai, NY, NY USA; 5 Icahn Institute for Data Science and Genomic Technology, Icahn School of Medicine at Mount Sinai, NY, NY USA; 6 Department of Psychiatry, Icahn School of Medicine at Mount Sinai, NY, NY USA; 7 Department of Genetics and Genomic Sciences, Icahn School of Medicine at Mount Sinai, NY, NY USA; 8 Center for Precision Medicine and Translational Therapeutics, James J. Peters VA Medical Center, Bronx, NY, USA; 9 Mental Illness Research Education and Clinical Center, James J. Peters VA Medical Center, Bronx, NY, USA; 10 Laboratory of Developmental Genetics, Rockefeller University, NY, NY USA; 11 Department of Psychiatry, UT Southwestern Medical Center, Dallas, TX, USA; 12 Department of Cardiology, Icahn School of Medicine at Mount Sinai, NY, NY USA; 13 Department of Diagnostic, Molecular and Interventional Radiology, Icahn School of Medicine at Mount Sinai, New York City, New York, USA; 14 The Cardiovascular Research Institute, Icahn School of Medicine at Mount Sinai, NY, NY USA; 15 Zuckerman Institute of Mind, Brain, and Behavior, Columbia University, NY, NY USA; 16 Howard Hughes Medical Institute, Icahn School of Medicine at Mount Sinai, NY, NY USA; 17 Senior author

## Abstract

Heightened activity in the orbitofrontal cortex (OFC), a brain region that contributes to motivation, emotion, and reward-related decision-making, is a key clinical feature of Major Depressive Disorder (MDD). However, the cellular and molecular substrates underlying this dysfunction remain unclear. Here, we performed cell-type-specific profiling of human OFC and unexpectedly mapped MDD-linked epigenomic features (including genetic risk variants) to non-neuronal cells, revealing significant glial dysregulation in this region. Characterization of MDD-specific chromatin loci further identified ZBTB7A—a transcriptional regulator of astrocyte reactivity—as an important mediator of MDD-related alterations. In rodent models, we found that Zbtb7a induction in astrocytes is both necessary and sufficient to drive stress-mediated behavioral deficits, cell-type-specific transcriptional/epigenomic signatures, and aberrant OFC astrocyte-neuronal communication in male mice—an established MDD risk factor. These findings thus highlight essential roles for astrocytes in OFC-mediated stress susceptibility, and identify ZBTB7A as a critical and therapeutically relevant regulator of MDD-related OFC dysfunction.

## eTOC

Fulton et al. identify the chromatin regulator ZBTB7A as a key driver of stress susceptibility via its actions in astrocytes of the orbitofrontal cortex. Using cross-species epigenomic profiling and rodent models, they show that astrocytic ZBTB7A reshapes neuronal activity and behavior following stress, revealing a glial-mediated epigenetic mechanism of depression vulnerability.

## INTRODUCTION

Major Depressive Disorder (MDD) is a leading cause of disability worldwide^1^ and is characterized by persistent negative mood, cognitive impairment, and anhedonia—the reduced capacity to experience pleasure, which is a symptom that is often resistant to standard antidepressant treatments^2–5^. While many prefrontal cortical areas have been implicated in affective disorders, the orbitofrontal cortex (OFC) has recently emerged as a key hub within MDD-relevant corticolimbic networks^2^. The OFC is responsible for reward processing and motivated behavior, both of which are critically disrupted in MDD patients^2^. Functional imaging studies have consistently identified OFC hyperactivation in MDD subjects, in contrast to the reduced activity often observed in other prefrontal regions^6–16^. Furthermore, several reports have shown that heightened activity of the OFC correlates with anhedonia severity, suicidality, treatment resistance, and pathogenic trajectories of depression^6–16^. Although these clinical observations point to a clear role for the OFC in MDD, the precise molecular and cellular mechanisms driving these functional changes remain poorly defined.

Stress exposure is a major risk factor for MDD^17^, and mounting evidence suggests that epigenetic processes are a key mechanism through which stressful experiences interact with genetic risk factors to promote depressive symptoms. Disease-related cellular phenotypes are often shaped by stimulus-induced transcription factors (TFs) that orchestrate gene expression programs in a cell-type-specific manner^18–21^. In stress-related disorders, these epigenetic processes can become maladaptive, rendering specific cell populations “primed” for exaggerated transcriptional responses to subsequent stressors. Here, we conducted gene expression and chromatin accessibility profiling of human postmortem OFC to characterize cell-type specific molecular drivers of MDD pathology. MDD-linked markers—including genetic risk variants—revealed significant glial dysregulation and identified ZBTB7A as a key TF enriched in MDD-specific active chromatin regions. ZBTB7A is a chromatin remodeling factor known for its context-dependent regulatory activity during inflammatory responses in diverse cell-types, but its functions in the central nervous system have not yet been characterized. To probe its function *in vivo*, we leveraged viral manipulations, cell-type-specific molecular profiling, and OFC neural recordings in rodent stress models. We show that astrocyte-specific ZBTB7A activity governs key transcriptional responses to stress, including inflammatory pathways and astrocytic homeostatic control of neuronal transmission, leading to increases in OFC activity and depression-like stress susceptibility in male mice. Together, our findings support a model in which ZBTB7A acts as an astrocyte-specific epigenetic regulator that links stress experience to OFC dysfunction and MDD-relevant stress susceptibility phenotypes^22–25^.

## RESULTS

### Molecular profiling of OFC identifies molecular signatures of human MDD

**Fig. 1.**
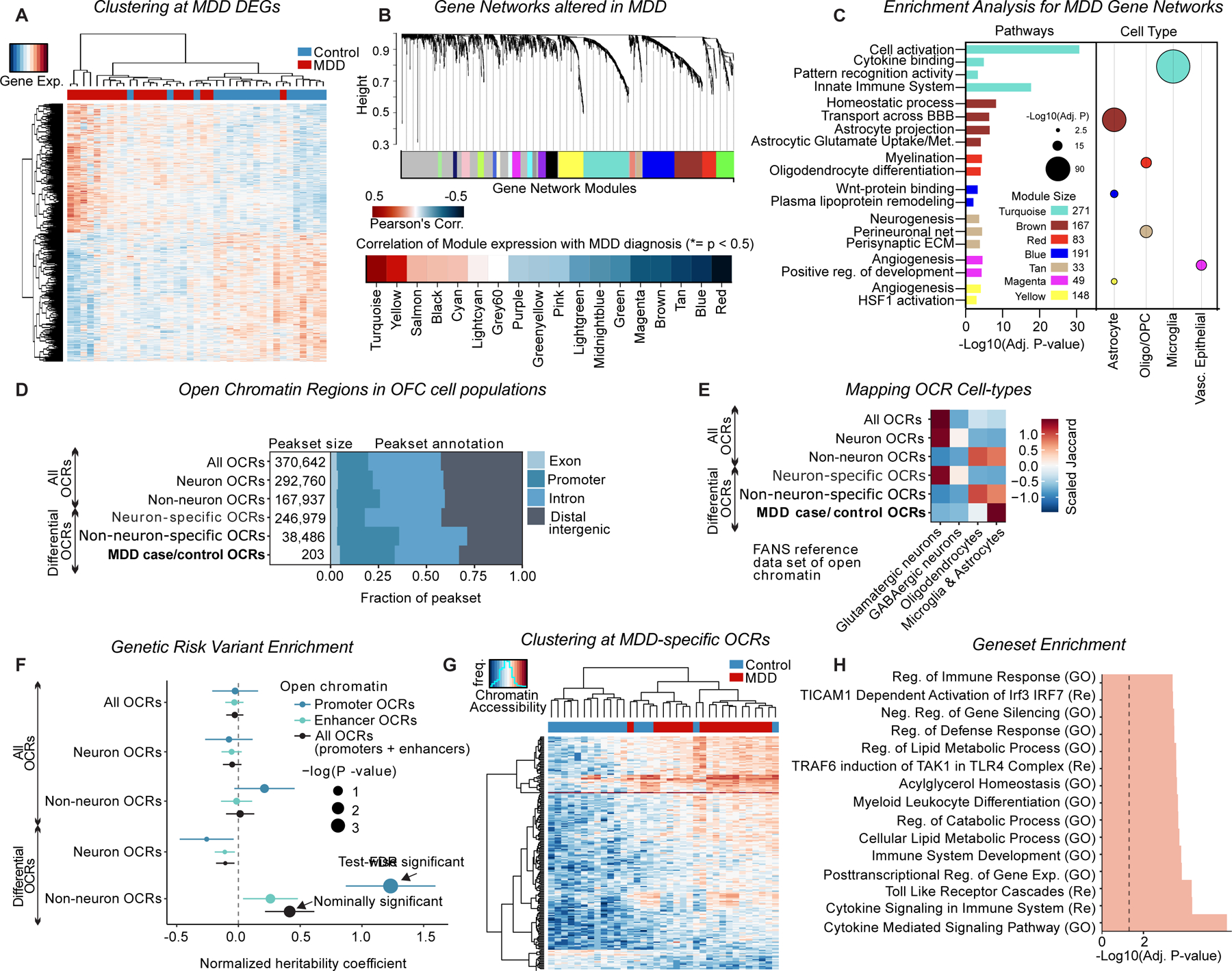
Transcriptomic and chromatin accessibility profiling identifies glial regulatory signatures of human MDD in OFC. (**A**) Clustering of MDD case and control samples at 1,412 differentially expressed genes (DEGs) (FDR < 0.1). (**B**) Dynamic tree cut graph visualizing the co-expressed gene networks identified in human RNA-seq by weighted gene correlation network analysis (WGCNA) [top] and heatmap depicting each network’s Pearson’s correlation with MDD diagnosis [bottom]. * indicates Adj. P <0.05 significant correlation. (**C**) Gene Ontology (GO) analysis and Cell-type Enrichment for genes in each significantly MDD-correlated network. (**D**) Number of detected Open Chromatin Region (OCR) sets stratified by genomic context. (**E**) Overlap of OCR sets with a reference study of lineage-specific brain open chromatin atlas. (**F**) Enrichment of MDD-linked genetic variants within OCR sets by LD-score regression. (**G**) Clustering of MDD case and control non-neuronal samples at 203 MDD-specific OCRs (rows). (**H**) Overlap between GO and Reactome Pathway (Re) gene sets with the set of 203 MDD-specific OCRs. Dashed line indicates FDR = 0.05.

To investigate gene expression changes associated with MDD in the OFC, we performed bulk RNA-seq on postmortem OFC tissues from 20 individuals with MDD and 19 demographically matched controls. While aberrant OFC neuronal activity is a key hallmark of MDD, both differential gene expression and weighted gene co-expression network analysis (WGCNA)^26^ identified widespread transcriptional changes that were associated with glial cell function and inflammatory responses, suggesting a key role for non-neuronal cell dysregulation in this brain region (Figure S1A–D, Figure 1A–1C). Individual genes altered in MDD were enriched for microglia activation (upregulated genes) and astrocytic homeostatic functions (downregulated genes) (Fig. S1D). MDD-linked gene networks mapped exclusively to glial cell-types and were involved in microglial immune response, astrocytic homeostatic regulation of glutamate and the perisynaptic net, and myelination pathways (Figure 1C).

To explore the regulatory landscape underlying these transcriptomic changes, we isolated neuronal and non-neuronal nuclei from the same cohort using Fluorescence-Activated Nuclear Sorting (FANS), followed by ATAC-seq (*A*ssay for *T*ransposase-*A*ccessible *C*hromatin followed by Sequencing). After extensive quality control, we analyzed chromatin accessibility across 70 high-quality libraries (Table S1, S2 and Figure S1E–N). We identified Open Chromatin Regions (OCRs), accounting for 4.79% of the genome in neurons and 2.65% in non-neuronal cells (Figure 1D), and matched these data to publicly available cell-type-specific ATAC-seq reference datasets^27^ (Figure 1E, Figure S1F). Given that the majority of disease-associated genetic variants lie in non-coding regulatory regions of the genome^20^, we calculated the enrichment of MDD-linked SNP heritability^28^ across cell-type specific OCRs (Figure 1F). MDD risk variants were significantly enriched in non-neuronal-specific open promoters, but not in any neuronal regions^29^ (Figure 1F), suggesting that active regulatory elements in OFC glial cells may mediate genetic risk for MDD.

Next, we performed differential chromatin accessibility analysis to identify putative *cis*-regulatory elements that are specific to MDD. Consistent with transcriptomic findings, we found 203 MDD-specific OCRs in non-neuronal samples, with none detected in neurons (Figure 1G, Figure S1N–P, Table S3). Genes regulated by these MDD-specific OCRs, such as Nuclear Corepressor 2 (*NCOR2)*—a negative regulator of astrocyte reactivity^30^—were significantly altered in MDD samples (Figure S1Q–R). Gene set enrichment analysis (GSEA)^31^ further revealed that MDD-specific OCRs were enriched for glial activation, NF-kB-induced inflammation, cytokine-mediated cascades, lipid metabolism, and toll-like receptor signaling^32–36^) (Figure 1H, Table S4). Together, these data define glial regulatory features in the human OFC that may underlie MDD-associated stress responses and pathophysiology^22–25^.

### Identification of ZBTB7A as a key TF regulating MDD-specific OCRs in OFC

**Fig. 2.**
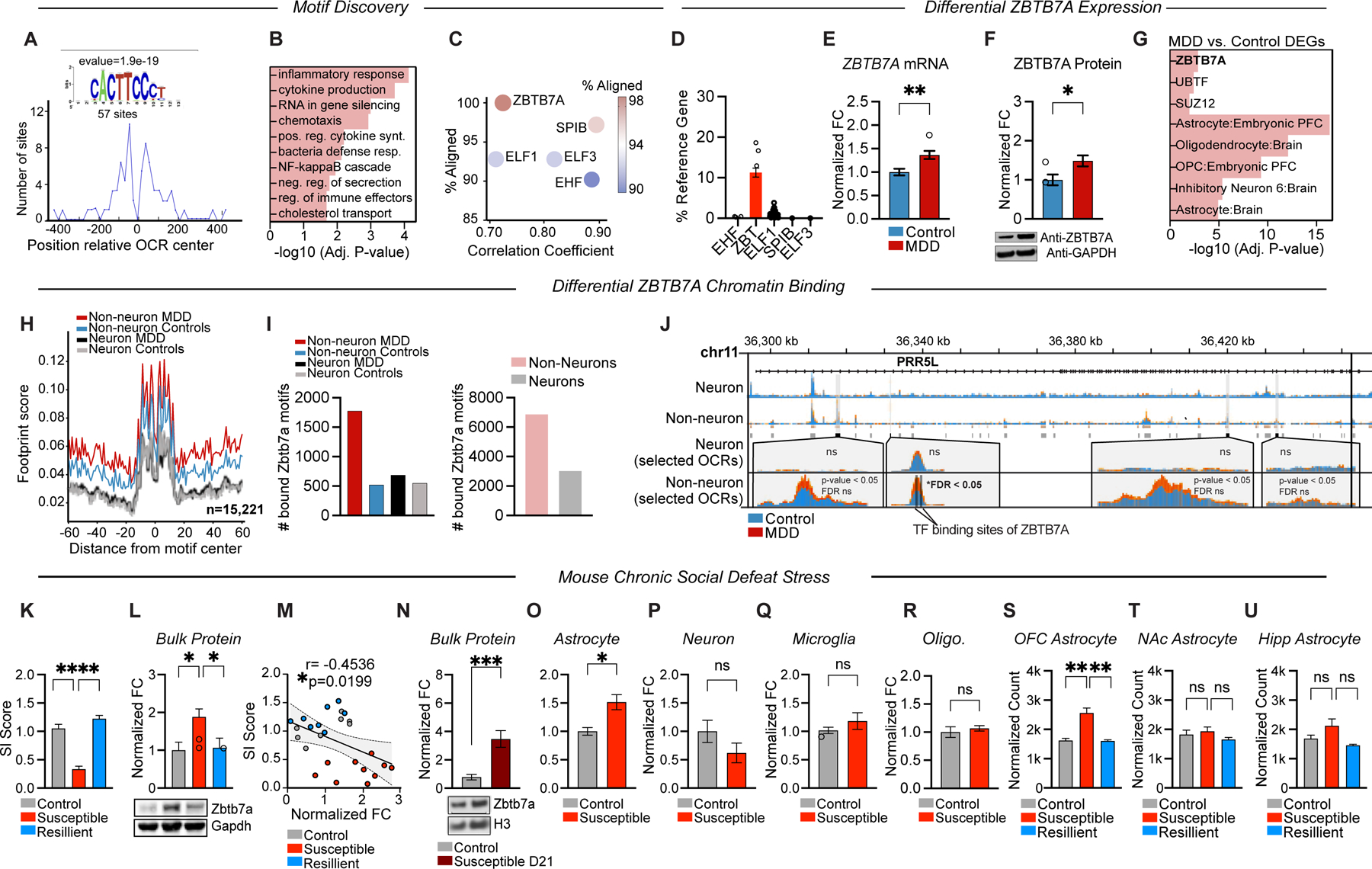
Identification of ZBTB7A as a key transcription factor regulating MDD-specific OCRs in OFC. (**A**) Distribution of the discovered motif enriched in MDD-specific OCRs. (**B**) Top 10 most significant GO terms for gene targets of OCRs containing the discovered motif. (**C**) Correlation coefficients and percent alignment between candidate TF motifs and the discovered motif. (**D**) Percent expression of candidate TF genes (CT value) over reference gene (*HPRT1*). “n.d.” indicates not detected. (**E**) *ZBTB7A* mRNA in OFC postmortem human tissues (MDD, n = 20 vs. control, n = 19) samples. (**F**) *Z*BTB7A protein in bulk OFC postmortem human tissues (MDD, n=15 vs. control, n=12) samples]. See western blot film scans in Figure S6B–C. (**G**) Enrichment of terms in CellMarker Augmented Database^24^ and ChEA ENCODE Consensus database^107^ for downregulated MDD DEGs. (**H**) Aggregated footprint scores across ZBTB7A sites in MDD or control nuclei populations. (**I**) Number of bound ZBTB7A sites detected exclusively in MDD or control neuronal vs. non-neuronal nuclei [right] and in all non-neuronal/neuronal nuclei [left]. (**J**) Representative traces of cell specific ATAC-seq signal overlapping *PRR5L* locus at four OCRs, with the only significantly dysregulated OCR between MDD and control (FDR<0.05) overlapping two ZBTB7A TFBS. (**K**) Social interaction (SI) ratio score for control (n = 8) vs. CSDS susceptible (n = 11) vs. CSDS resilient mouse (n = 9) groups (**L**) Protein expression of Zbtb7a in mouse OFC bulk tissues: control vs. susceptible vs. resilient groups. See raw western blot film scans in Figure S6D–E. (**M**) Correlation between SI score and Zbtb7a protein expression. (**N**) Zbtb7a protein expression in mouse OFC bulk tissues, control (n=11) vs. susceptible (n=14) at 21 days post-CSDS. See western blot film scans in Figure S6F–G. (**O-R**) *Zbtb7a* mRNA expression in MACs-isolated cells from chronically stressed OFC mouse tissues vs. control, n=4/group: (**O**) astrocytes, (**P**) neurons, (**Q**) microglia, and (**R**) immature oligodendrocytes. (**S-U**) FPKM values for *Zbtb7a* mRNA in CSDS astrocyte-specific TRAP-seq data set [GSE139684], n=3–5/group: (**S**) OFC (**T**) Nucleus Accumbens (**U**) Hippocampus. For all graphs, data are mean ± SEM. One-way ANOVA with Tukey’s multiple comparisons test: ∗∗∗∗p < 0.0001, ∗∗∗p < 0.001, ∗∗p < 0.01, ∗p < 0.05; ns, not significant. Full statistics information is provided in the Supplemental Table S7_stats.

We next conducted TF motif discovery and enrichment analysis^37^ within MDD-specific OCRs to identify upstream regulators. A single motif was significantly enriched, with 57 motif occurrences in 202 OCRs (Figure 2A). Gene ontology motif (GO-MO) analysis^38^ linked this motif to cellular inflammatory signaling (Figure 2B), paralleling GO results from the full set of MDD-specific OCRs (Figure 1H). Among the top five TF candidates, ZBTB7A was the only one found to be both expressed in the human brain and significantly dysregulated in MDD (Figure 2C–D, S2A). ZBTB7A (also known as LRF, Pokemon, or FBI-1), is a pleiotropic DNA-binding protein with context-dependent chromatin regulatory roles^39^. ZBTB7A has previously been shown to coordinate chromatin structure that is necessary for NF-kB dependent gene expression in various cancers (notably gliomas) and other inflammatory conditions^40^; however its functions in the brain and/or potential contributions to psychiatric disease have not yet been explored.

In our human cohort, ZBTB7A mRNA and protein were significantly upregulated in MDD vs. control OFC (Figure 2E–F, S2B). ZBTB7A gene targets were also enriched among MDD DEGs identified in our RNA-seq analyses, as well as in prior MDD transcriptomic studies^41^, where ZBTB7A was found to be upregulated in the frontal cortex, but not in other non-cortical regions, such as the hippocampus (Figure 2G). Footprinting analysis^42^ on our ATAC-seq data revealed a 43.8% higher number of bound ZBTB7A sites in non-neuronal *vs.* neuronal cells, with a 3.4-fold increase in occupied ZBTB7A sites identified in non-neuronal MDD samples compared to controls––making it one of the top five most differentially bound TFs genome-wide (Figure 2H,I). One illustrative example is the *PRR5L* (proline rich 5 like gene) locus, a MDD biomarker gene^43,44^, which harbors a MDD-specific OCR that overlaps two ZBTB7A binding sites (Figure 2J).

To investigate potential roles for ZBTB7A in MDD-related phenotypes, we turned to an etiologically relevant mouse model for the study of depression in male mice––the chronic social defeat stress (CSDS) paradigm––which induces robust behavioral deficits that are similar to those observed in human patients, including social avoidance and reward insensitivity^45^. Importantly, CSDS also models natural variation in stress vulnerability, as ~30% of wild-type mice exposed to this paradigm do not develop social avoidance or other stress-related phenotypes and are thus termed stress-resilient (*vs.* stress-susceptible). In CSDS mice, Zbtb7a protein expression was persistently upregulated in bulk OFC tissues in stress-susceptible (but not resilient or control) mice at 48 hours and 21 days following CSDS (Figure 2K–N, S2C).

ZBTB7A was recently shown to act as a master transcriptional regulator of astrocyte inflammatory activation in a CRISPR-screen using human IPSC-derived cells^46^. Therefore, to investigate potential cell-type specific patterns of *Zbtb7a* upregulation in stress-susceptible mice, we used Magnetically Activated Cell Sorting (MACs) to isolate astrocytes, neurons, oligodendrocytes, and microglia (Figure S2D–G) from a separate CSDS cohort. *Zbtb7a* mRNA was selectively increased in astrocytes following CSDS, with no significant changes in other cell-types (Figure 2O–R). Immunostaining confirmed that Zbtb7a protein is robustly expressed in mouse CSDS OFC astrocytes, with minimal co-localization with microglial markers (Figure S2H–I). Interestingly, in control mice, *Zbtb7a* expression was higher in neurons compared to astrocytes, consistent with human single-cell sequencing studies in healthy tissues^47,48^, indicating a potential stress-induced shift in expression patterns (Figure S2J). Astrocyte-specific Translating Ribosome Affinity Purification coupled to sequencing (TRAP-Seq) data across three different brain regions from CSDS mice further confirmed that active translation of *Zbtb7a* mRNA is robustly upregulated in frontal cortical (but not hippocampal or Nucleus Accumbens) astrocytes of stress-susceptible mice, suggesting regional specificity for Zbtb7a upregulation in astrocytes after stress (Figure 2S–U).

Consistent with a potential role for astrocytic ZBTB7A in stress susceptibility, MDD DEGs and MDD-linked non-neuronal promoters were enriched for both ZBTB7A regulation and astrocyte-related pathways (Figure 2G, Figure S2K). To determine whether ZBTB7A upregulation impacts MDD-specific OCR gene targets in human astrocytes, we used a lentivirus to overexpress (OE) ZBTB7A in human primary cortical astrocytes, which resulted in a robust induction of MDD-specific OCR target genes, as well as other key MDD DEGs within the NF-kB pathway (Figure S2L–O). In both human and mouse-derived cortical cultured astrocytes, lipopolysaccharide (LPS)-induced inflammatory stimulation also significantly increased *ZBTB7A/Zbtb7a* expression, further linking this chromatin regulator to astrocytic cellular reactivity pathways (Figure S2P, Q).

### Astrocytic ZBTB7A overexpression is sufficient to induce stress susceptibility

**Fig. 3.**
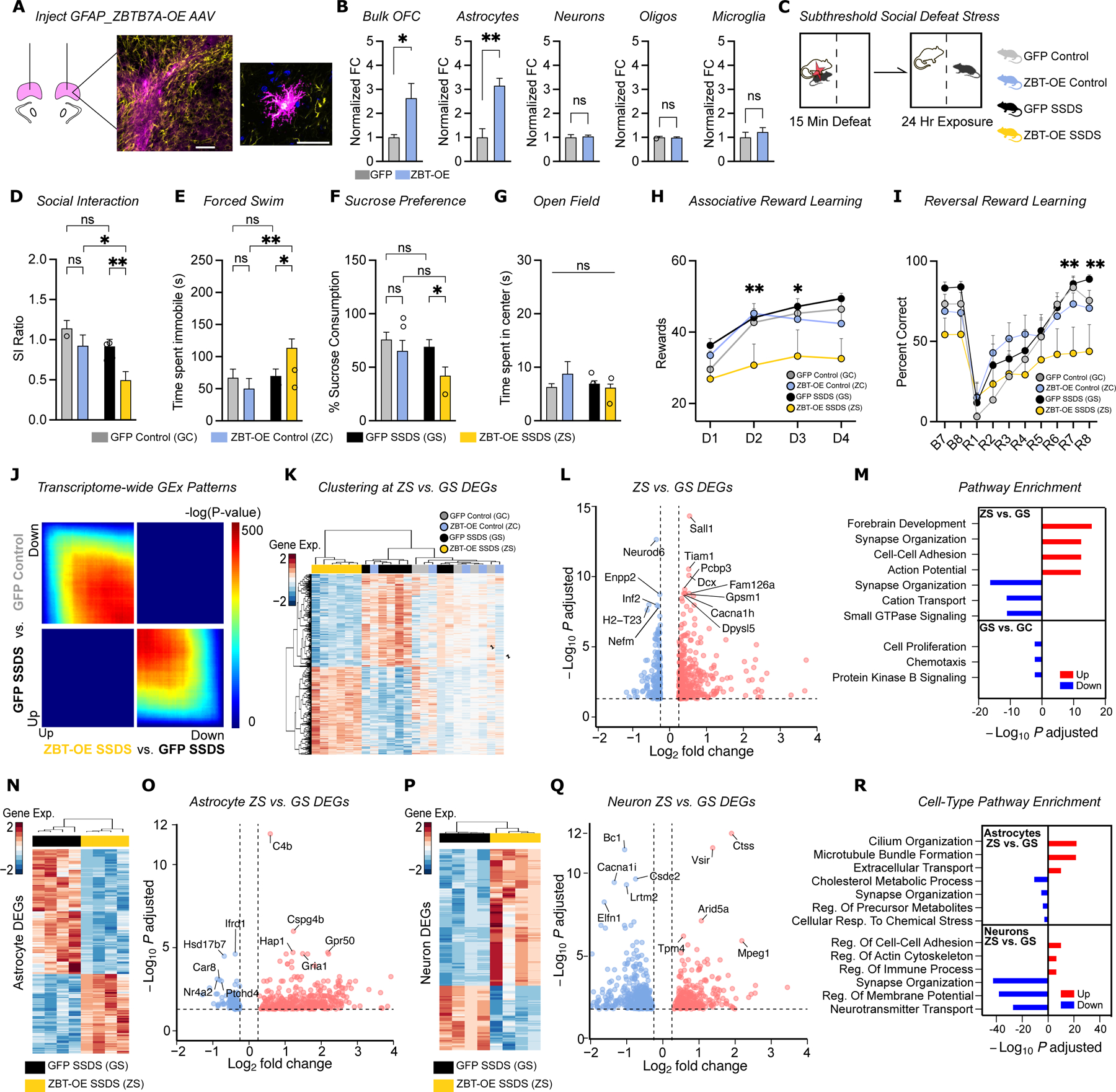
Astrocyte-specific ZBTB7A OE in mouse OFC is sufficient to induce behavioral and molecular signatures of stress susceptibility. (**A**) Schematic of AAV6-GFAP-ZBTB7A-OE injection into OFC with example histology of virus expression. (**B**) *Zbtb7a* mRNA in bulk OFC tissue, MACs-isolated astrocytes, neurons, oligodendrocytes, and microglia from AAV6-GFAP-ZBT-OE transduced OFC tissues, n=2–4/group. (**C**) Schematic of experimental groups resulting from SSDS paradigm performed after AAV injection into OFC (**D**) Social interaction ratio. (**E**) Time spent immobile in Forced Swim test. (**F**) Percent sucrose consumed during the Sucrose Preference Test. (**G**) Time spent in center during open field test. (**H**) Performance in cue-reward association task. “D” indicates day of test. 3-way ANOVA, Virus x Stress. Stars denote significant Sidak’s MC test between groups per test day, see Figure S3C. (**I**) Percent correct trials in reversal learning paradigm. “B” indicates Baseline Day, “R” indicates Reversal phase day. 3-way ANOVA, Test Day x Virus. Stars denote significant Sidak’s MC test between groups per test day, see Figure S3F. (**J**) RRHO comparing gene expression overlaps, where each pixel color represents the hypergeometric test overlap between differential transcriptomes. (**K**) Clustering at 1,929 DE genes between ZBT-OE SSDS and GFP SSDS. (**L**) Volcano plot of Bulk RNA-seq DEGs. (**M**) GO analysis for up/downregulated bulk DEGs in ZBT-OE SSDS vs. GFP SSDS, and GFP SSDS vs. GFP control. (**N**) Clustering at 715 DEGs between ZBT-OE SSDS vs. GFP SSDS astrocytes (n = 4/group). (**O**) Volcano plot depicting astrocyte DEGs (FDR < 0.1). (**P**) Clustering at 1,191 DEGs between ZBT-OE SSDS vs. GFP SSDS neurons (n = 4/group). (**Q**) Volcano plot depicting neuron DEGs (FDR < 0.1). (**R**) GO analysis for up/downregulated DEGs (FDR < 0.1) in astrocytes and neurons between ZBT-OE SSDS vs. GFP SSDS. All data graphed as means ± SEM. Data analyzed with 2-way or 3-way ANOVA with Sidak’s multiple comparisons test: ∗∗∗∗p < 0.0001, ∗∗∗p < 0.001, ∗∗p < 0.01, ∗p < 0.05; ns, not significant. Full statistics information is provided in the Supplemental Table S7_stats.

Based on our human and mouse data, we hypothesized that overexpression of ZBTB7A in OFC astrocytes may promote increased stress susceptibility. To test this, we packaged a GFAP promoter-driven ZBTB7A overexpression (ZBT-OE) construct (vs. an empty vector GFP control) into an AAV6 virus^38^ and confirmed robust, astrocyte-specific ZBTB7A OE in transduced mouse OFC tissues (Figure 3A, B and Figure S3A, B). Mice were transduced either AAV-GFAP-ZBT-OE or GFP virus intra-OFC and subjected to subthreshold social defeat stress (SSDS), a one-day acute social defeat paradigm that does not elicit behavioral deficits in normal wild-type animals^49^ (Figure 3C). Following SSDS, as expected, GFP mice showed no significant differences between SSDS groups and controls. In contrast, ZBT-OE SSDS mice exhibited heightened social avoidance [i.e., a lower Social Interaction (SI) Ratio] compared to GFP SSDS animals, a phenotype that is commonly observed in chronically stressed mice (Figure 3D). ZBT-OE SSDS mice also exhibited increased immobility in the forced swim test and reduced sucrose consumption in the sucrose preference test, consistent with helplessness and anhedonia-like phenotypes (with no differences observed in anxiety-like behavior in the open field test) (Figure 3E–G). ZBT-OE also induced deficits in OFC-dependent reward learning behaviors, which reflect changes in hedonic drive in both rodents and humans. ZBT-OE SSDS mice showed significant deficits in cue-reward association, instrumental reward learning, and reversal reward learning tasks^50^, paralleling OFC dysfunction observed in MDD patients^51^ (deficits that can be reversed with antidepressant treatments) (Figure 3H–I, S3C–F). Interestingly, ZBT-OE alone (without stress exposure) did not consistently lead to behavioral effects, which is in agreement with previous reports showing that ZBTB7A acts primarily to transduce cellular signals into gene expression changes by regulating chromatin accessibility for secondary TFs^40^. Without an additional stimulus to induce NF-kB activation, for example, ZBTB7A OE may not be sufficient to induce a full cellular response on its own, but may prime chromatin states for heightened transcriptional responses to subsequent stressful experiences^40^.

### Astrocytic ZBTB7A overexpression promotes stress-mediated molecular profiles

To explore the molecular correlates of increased stress susceptibility following ZBT-OE, we next performed bulk RNA-seq on virally transduced OFC tissues from all four groups. We employed Rank-Rank Hypergeometric Overlap (RRHO)^52^ analysis to correlate global gene expression patterns between groups, as well as DEG analysis to identify gene pathways that are impacted by ZBT-OE and SSDS exposure. Both analyses showed that ZBT-OE reverses typical gene expression responses to acute stress *vs.* GFP mice, while unstressed ZBT-OE controls showed only mild changes (Figure 3J–K, Figure S3G–H). Top DEGs between ZBT-OE SSDS and GFP SSDS included known regulators of astrocyte morphological remodeling, reactive astrogliosis, and inflammatory stress responses*: Tiam1*^53^*, Pcbp3*^54^*, Enpp2*^55^, and *H2-T23*^56^ (Figure 3L). Overall, DEGs were involved in pathways related to astrocyte activation, such as regulation of ionic transport, cellular adhesion, and synaptic organization (Figure 3M). Although there were only a small number of DEGs observed between GFP controls and GFP SSDS mice (19 genes, FDR<.1), they reflected changes in well-characterized MDD/stress-related genes, such as an increase in the resilience-related gene *Fkbp5*^57,58^ and a decrease in the inflammatory cytokine *Cxcl12*^59^. Notably, inflammatory gene sets were significantly downregulated in GFP SSDS vs. GFP control mice, suggesting that normal behavioral resilience to acute stress may involve pro-adaptive transcriptional responses (Figure S3I). Accordingly, flow cytometry confirmed that ZBT-OE in astrocytes significantly increased functional markers of neuroinflammation after SSDS, including higher percentages of activated microglia (with no differences observed in overall cell numbers) (Figure S3J).

To define cell-type-specific effects of this manipulation, we isolated astrocytes from the two SSDS groups (ZBT-OE vs. GFP), and performed ATAC-seq and RNA-seq (Figure S3K). ZBT-OE + SSDS robustly altered chromatin accessibility patterns (6,094 differentially accessible regions) at loci linked to ZBTB7A gene targets and enriched for pathways involved in ion homeostasis, synaptic regulation, and inflammatory signaling (Figure S3L–O). Key DEGs included upregulation of *C4b*, a protein that facilitates astrocyte phagocytosis during immune activation^60^; *Gpr50* (a genetic risk factor for MDD^61,62^); and *Cspg4b*, a hub gene previously identified in stress-susceptible gene networks^63^ (Figure 3N,O). We also detected downregulation of the anti-inflammatory factor *Nr4a2*^64^, as well as *Hsd17b7,* a hallmark response of reduced neuronal trophic support in disease-associated astrocytes^65^. RNA-seq on MACS-isolated neurons from the same mice revealed upregulation of cellular stress response genes, as well as reduced expression of pathways involved in neurotransmitter transport and synaptic organization, suggesting that ZBT-OE in astrocytes may influence stress susceptibility through cell non-autonomous effects on OFC neuronal function (Figure 3P–R).

### Astrocytic ZBTB7A overexpression disrupts OFC neuronal activity patterns

**Fig. 4.**
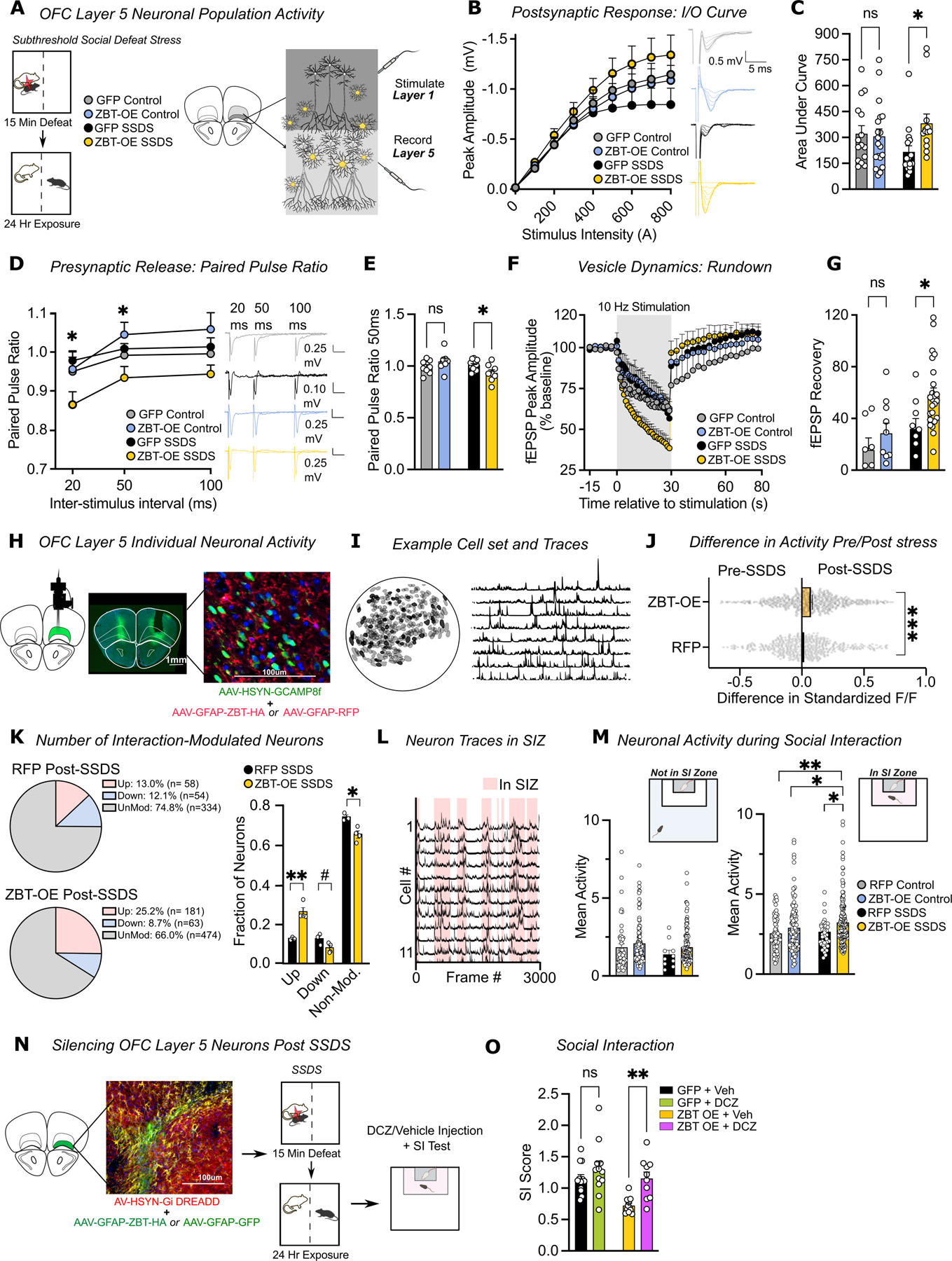
Astrocyte-specific ZBTB7A overexpression disrupts OFC neuronal activity patterns in response to mild stress. (**A**) Schematic of experimental timeline with subthreshold stress paradigm performed after AAV6 injection into OFC, followed by slice electrophysiological recordings. (**B**) Input-output (I-O) curve of fEPSPs in response to stimuli. 3-way ANOVA, Interaction of Stimulus Intensity x Virus x Stress. (**C**) Individual values for (I-O) curve, area under curve (A.U.C). (**D**) fEPSP Paired-pulse ratio (PPR) 3-way ANOVA, Interaction of Stress x Virus. (**E**) Individual values for PPR at 50ms. (**F**) Percent change in fEPSP amplitude from baseline during 10Hz Stimuli train. 3-way ANOVA, Stimulus x Virus, Stress x virus. (**G**) Individual values for fEPSP recovery from rundown measured 1s after the simulation train. (**H**) Lens placement and IHC validation of hsyn-gCAMP8f (green, neurons) and GFAP-ZBT OE (red, astrocytes) in OFC Layer 5. (**I**) Example cell set and calcium traces. (**J**) Difference in standardized deltaF/F for neurons Pre- vs. Post-SSDS. (**K**) Right: Percentage of OFC neurons upmodulated by entry into social interaction zone with aggressor mouse. Left: Fraction of neurons significantly modulated by social interaction, stratified by modulation type for ZBT-OE SSDS vs. RFP SSDS. (**L**) Activity traces for example SI upmodulated neurons in ZBT-OE subject post-SSDS. (**M**) Mean activity of modulated neurons while [Right] mouse was outside the social interaction zone and [Left] while mouse was inside the social interaction zone. (**N**) IHC validation of AAV-hsyn-hM4D(Gi)-mCherry (in red, neurons) and GFAP-ZBT OE (in green, astrocytes) with GFAP (yellow) and DAPI (blue), and experimental scheme of chemogenetics experiment, in which SSDS is performed on a cohort of mice expressing hM4D(Gi)-mCherry (+/−) ZBT OE, (+/−) DCZ. (**O**) Social interaction with chemogenetic inhibition. All data graphed as means ± SEM. Data analyzed with 2-way or 3-way ANOVA with Sidak’s multiple comparisons test: ∗∗∗∗p < 0.0001, ∗∗∗p < 0.001, ∗∗p < 0.01, ∗p < 0.05; ns, not significant. Full statistics information is provided in the Supplemental Table S7_stats.

Astrocytes have a direct impact on neurotransmission through homeostatic modulation of synaptic communication^66^, and reactive astrocytes can promote aberrant neuronal activity in surrounding neuronal networks. Given that astrocyte-specific ZBT-OE + SSDS induces chromatin rearrangements and gene expression changes that are linked to synaptic dysregulation, we next asked whether this manipulation increases OFC neuronal population activity—a hallmark clinical feature of MDD. To address this, we recorded *ex-vivo* field excitatory postsynaptic potentials (fEPSP) from OFC Layer 5 neuronal populations (comprising the major output cell layer of OFC) in ZBT-OE *vs.* GFP (+/−) SSDS mice (Figure 4A). ZBT-OE SSDS mice exhibited increased fEPSP amplitudes in response to Layer 1 stimulation, increased paired-pulse ratios (PPR), and altered synaptic vesicle dynamics, including faster rundown and recovery during 10Hz stimulation trains^67^ – suggestive of increased pre- and postsynaptic efficiency relative to GFP SSDS and unstressed controls (Figure 4B–G and Figure S4A–B). Collectively, these data are consistent with observations from human MDD OFC imaging studies and demonstrate that increased ZBTB7A expression in astrocytes potentiates OFC neuronal population activity in response to acute stress, with GFP-expressing mice instead displaying reductions in OFC activity that may underlie behavioral resilience observed in this group.

To explore how astrocytic ZBT-OE affects individual OFC neuronal responses *in vivo* during relevant behavioral activity, we performed microendoscopy calcium imaging of Layer 5 OFC neurons in ZBT-OE *vs.* empty vector control mice during social interaction testing before and after SSDS exposure (Figure 4H–I). We co-injected our GFAP-driven ZBT-OE virus (*vs.* an empty GFAP-RFP control) with AAV9-hsyn-Gcamp8f (a highly sensitive and ultra-fast calcium indicator^68^) intra-OFC and implanted a gradient refractive index (GRIN) lens above OFC Layer 5 to visualize neuronal calcium transients (Figure S4C).

Prior to SSDS, we did not observe significant differences in individual OFC neuron activity between the two viral groups, either during baseline home cage recordings or during a social interaction test, consistent with our previous results in unstressed control mice (Figure S4D, Figure 4M). In contrast, comparing the activity of the same individual cells pre- *vs.* post-SSDS revealed that neurons in ZBT-OE mice had increased mean activity during the SI test following exposure to SSDS compared to RFP mice (Figure 4J, Figure S4E). To investigate how ZBT-OE affects OFC encoding of interactions with an aggressor mouse, we tracked the activity of individual OFC Layer 5 neurons while the mice moved between the social interaction zone, and identified neurons that were significantly modulated by social investigation using permutation analysis (Figure 4K–L, Figure S4F–H). ZBT-OE mice had a significantly higher percentage of neurons that were significantly modulated by social interaction, with 75.0% of these cells being upmodulated by aggressor interaction in ZBT-OE vs. 50.5% in GFP. Furthermore, the overall mean activity and correlation synchrony of OFC neuronal firing was significantly higher in ZBT-OE mice *vs.* RFP controls during interaction with the aggressor, suggesting that this manipulation in astrocytes may actively shape local network dynamics to influence social behavior (Figure 4M , Figure S4I, J).

Finally, to test if these observed increases in neuronal OFC activity represent a functional link between ZBTB7A-mediated molecular changes and stress susceptibility, we used a chemogenetic approach with hM4Di DREADDs to silence Layer 5 OFC neurons in the context of ZBT-OE in astrocytes and SSDS (note that for this experiment, all mice went through the SSDS exposure) (Figure 4N, S4K). Prior to the social interaction assay, half of each viral group was injected with either vehicle or the DREADD agonist Deschloroclozapine^69^ (DCZ) (which we confirmed had no effect on previously observed social avoidance phenotypes in ZBT-OE SSDS mice without DREADD expression (Figure S4L–M)]. In vehicle injected mice, ZBT OE + SSDS again resulted in reduced social interaction behavior, however, chemogenetic inhibition of OFC neurons rescued this social avoidance phenotype (Figure 4O), demonstrating that increased OFC activity caused by ZBT-OE in astrocytes drives stress-related behavior. Together with our neural recording data, these findings indicate that astrocytic ZBTB7A upregulation promotes stress vulnerability through dysregulation of OFC neuronal activity, which is consistent with clinical reports of astrocyte dysfunction and heightened OFC activity in human MDD^10^.

### Astrocytic Zbtb7a knockdown reverses stress-induced phenotypes

**Fig. 5.**
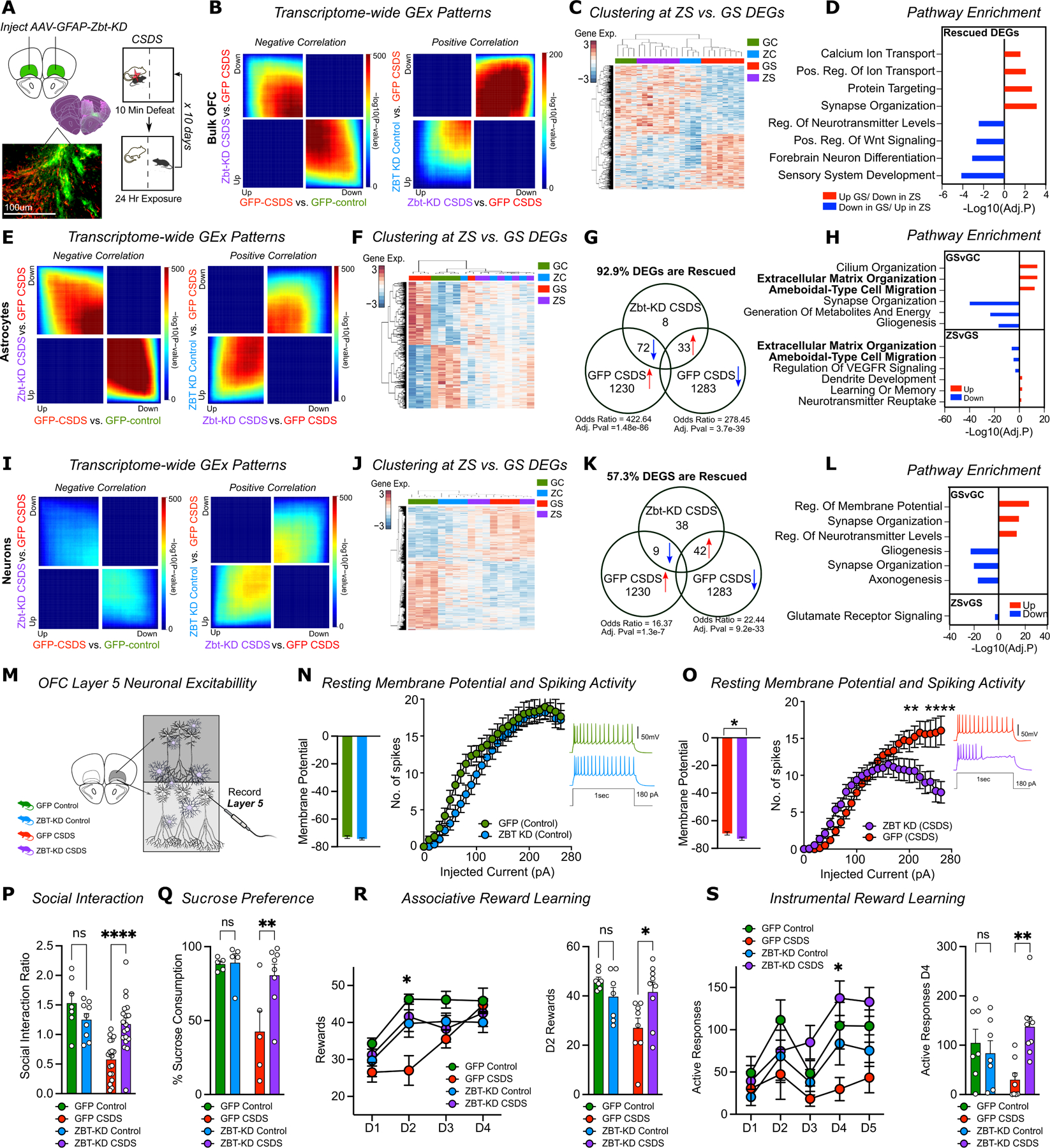
Knockdown of Zbtb7a in mouse OFC astrocytes reverses molecular, synaptic, and behavioral signatures of chronic stress. (**A**) CSDS paradigm performed after AAV-GFAP-ZBT-KD-GFP vs. negative miR-GFP control injection into OFC, with example IHC viral expression. (**B**) RRHO comparing gene expression overlaps in bulk OFC tissue. (**C**) Clustering of groups at 1,583 DEG (FDR < 0.1) for GFP stress vs. GFP control in bulk OFC. (**D**) GO analysis for genes rescued by Zbt-KD stress vs. GFP-stress. (**E**) RRHO comparing gene expression overlaps in MACS-isolated astrocytes. (**F**) Clustering at 2,673 DEGs (FDR < 0.1) between GFP stress vs. GFP control in MACS-isolated astrocytes. (**G**) Venn-diagram and odds ratio tests of the overlap between DEGs in astrocytes between Zbt-KD stress vs. GFP stress and GFP stress vs. GFP control. (**H**) GO analysis for up/downregulated DEGs in GFP-stress vs. GFP control and Zbt-KD stress vs. GFP-stress. (**I**) RRHO comparing gene expression overlaps in neurons. (**J**) Clustering of groups at 2,540 DEGs (FDR < 0.1) between GFP stress vs. GFP control in neurons. (**K**) Venn-diagram and odds ratio test of the overlap between DEGs in neurons between Zbt-KD stress vs. GFP stress, and GFP stress vs. GFP control. (**L**) GO analysis for up/downregulated DEGs in GFP-stress vs. GFP control and Zbt-KD stress vs. GFP-stress. (**M**) Patch clamp electrophysiology recording scheme. (**N-O**) Resting membrane potential and depolarization-evoked spiking for control and stress groups. (**P**) Social interaction. (**Q**) Sucrose Preference Test. (**R**) Right: Associative Learning task. “D” = Day of task. 3-Way ANOVA, main effect of Test Day x Stress. Left: Individual values for Day two of task shown on right. (**S**) Right: Instrumental reward learning task on FR1 schedule. 3-Way ANOVA, main effect of Virus x Stress]. Left: Individual values for Day four of task shown on right. All data graphed as means ± SEM. Data analyzed with 2-way or 3-way ANOVA with Sidak’s multiple comparisons test: ∗∗∗∗p < 0.0001, ∗∗∗p < 0.001, ∗∗p < 0.01, ∗p < 0.05; ns, not significant. Full statistics information is provided in the Supplemental Table S7_stats.

To test whether stress-induced Zbtb7a upregulation in mouse OFC astrocytes is necessary for the molecular and behavioral outcomes of chronic stress, we packaged a Zbtb7a-targeting microRNA (Zbt-KD) or scrambled control into an AAV6 vector under the GFAP promoter and confirmed selective knockdown in OFC astrocytes (Figure S5A–E)^38^. To define the molecular impact of Zbtb7a KD (Zbt-KD) in the context of chronic stress, we performed RNA-seq on virally-transduced tissues from a cohort of CSDS animals (GFP Control, Zbt-KD Control, GFP CSDS, and Zbt-KD CSDS) (Figure 5A). Strikingly, RRHO analyses showed that Zbt-KD largely reversed the transcriptomic signature of CSDS, shifting gene expression towards control-like patterns (Figure 5B). In line with previous reports indicating that Zbtb7a promotes NF-kB activation, GSEA analysis showed that Zbt-KD downregulates inflammatory gene sets induced by chronic stress, with cytokine production being the most significantly downregulated gene set (Figure S5F). Unsupervised clustering of 1,583 DEGs at FDR <0.1 between GFP CSDS and controls showed that Zbt-KD CSDS samples exhibit an intermediate expression profile that clustered with unstressed control animals (Figure 5C). Odds ratio analysis revealed significant rescue of stress-related genes: 37.8% of upregulated (Adj.pval = 4.2e-104), and 50.5% of downregulated genes (Adj.pval = 1.2e-209) (Figure S5G). Rescued pathways included synaptic organization, neurotransmitter regulation, and ionic maintenance – key astrocytic homeostatic functions disrupted by stress (Figure 5D).

To dissect cell-type-specific effects, we performed ATAC-seq and RNA-seq on MACs-isolated astrocytes from Zbt-KD vs. GFP groups (+/−) CSDS (Figure S5H–O). Zbt-KD rescued nearly half of the chromatin accessibility changes induced by chronic stress (42.3%, of up events, Adj. Pval = 2e-89 and 65.5% of down events, Adj.Pval = 0e+00) (Figure S5K). Promoters with altered accessibility in CSDS astrocytes were enriched for Zbtb7a targets, as were promoters that closed by Zbt-KD (Figure S5J). Notably, more than 50% of chromatin regions closed in CSDS overlapped with those altered by ZBT-OE SSDS mice (56.4%, Adj.Pval = 4e-152), and ~36% of these were reversed by Zbt-KD (35.9%, Adj.Pval = 5e-208) (Figure S5L). Pathway enrichment analysis of rescued OCRs identified processes related to astrocyte reactivity, including ion homeostasis, ECM alterations, and cellular morphogenesis (Figure S5O).

RNA-seq on the same astrocytes indicated that Zbt-KD in chronically stressed mice reverses transcriptome-wide gene expression patterns in astrocytes compared to GFP stress animals (Figure 5E). ~93% (105/113) of DEGs (FDR<0.1) between the two CSDS groups were rescued, including *Dusp6*^41^, a gene implicated in stress-induced behavioral responses (Figure 5F). Chronic stress downregulated key astrocytic functions (e.g., metabolic homeostasis, ionic transport, synaptic support) while upregulating reactivity-related genes (e.g., cell motility, morphological remodeling)—all pathways that were also rescued by Zbtb7a KD (Figure 5H). Notably, Zbt-KD restored expression and chromatin accessibility of *Slc1a2* (also known as *Eaat2)*, the astrocyte-specific glutamate clearance transporter which modulates neuronal excitability and has consistently been shown to be downregulated following chronic stress^70,71^, suggesting that astrocytic Zbtb7a may impact synaptic excitability through altered glutamate clearance (Figure S5P).

To assess downstream neuronal consequences, we performed RNA-seq on MACs-isolated OFC neurons from this same cohort. Zbt-KD also induced a moderate reversal of gene expression changes induced by CSDS in neurons, including genes involved in glutamate transmission and synaptic function, further supporting a role for astrocytic Zbtb7a in modulating neuron-glia interactions under stress (Figure 5I–L).

To determine if the observed molecular changes in Zbt-KD CSDS affect intrinsic neuronal properties, we performed *ex-vivo* patch-clamp electrophysiology recordings in Layer 5 OFC neurons. GFP CSDS neurons had significantly more depolarized resting membrane potentials vs. Zbt-KD CSDS animals (Figure 5M–O). In addition, OFC neurons in GFP CSDS mice fired more action potentials in response to depolarizing current injections vs. Zbt-KD CSDS neurons, indicating that astrocyte-specific Zbt-KD is sufficient to reduce the intrinsic excitability of OFC neurons in the context of CSDS; note that unstressed control animals showed no differences between the viral groups (Figure 5M–O). We observed no changes in input resistance or EPSP decay kinetics between the viral groups for either control or CSDS mice (Figure S5Q–S). Intriguingly, Zbt-KD CSDS neurons had lower PPR compared to GFP CSDS neurons, suggestive of a potential compensatory adaptation affecting presynaptic vesicle release (Figure S5T, U).

Finally, we assessed whether astrocytic Zbtb7a upregulation is required for behavioral vulnerability to chronic stress in a separate cohort of Zbt-KD *vs.* GFP (+/− CSDS) mice. Astrocyte-specific Zbt-KD in OFC was sufficient to fully rescue CSDS-induced social avoidance and sucrose preference deficits, with no significant changes observed in Zbt-KD control mice (Figure 5P–Q). Zbt-KD + CSDS mice also performed significantly better in two different measures of reward sensitivity: the associative and instrumental reward learning tasks (Figure 5R–S, S5V). Whereas chronically stressed mice learned the reward contingencies of these tasks significantly slower than controls, we observed a robust increase in the number of rewards earned for the Zbt-KD CSDS mice compared to GFP CSDS animals (with no differences in locomotor activity) (Figure 5R–S, Figure S5V). Together, these results demonstrate that Zbtb7a induction in OFC astrocytes following chronic social stress is a key epigenetic mechanism promoting behavioral stress responsivity, including social avoidance and reward insensitivity.

## DISCUSSION

### Cross-species evidence for glial dysregulation in MDD OFC and a key role for ZBTB7A in stress susceptibility

Overall risk for MDD arises from complex interactions between genetic factors and environmental stressors that alter corticolimbic function^72^. Epigenetic mechanisms, including chromatin remodeling, play a critical role by shaping brain responses to stress, thereby influencing individual susceptibility in a manner that is highly specific to the distinct molecular context of a given brain region or cell-type. Here, we identify ZBTB7A as a key transcription factor enriched in MDD-linked chromatin loci in the human OFC and demonstrate its critical role in astrocyte-mediated stress susceptibility.

ZBTB7A is a multifunctional chromatin remodeler, which can bind DNA and recruit co-repressor or co-activator complexes to its target loci. Its C-terminal zinc fingers confer DNA-binding specificity, while its BTB/POZ domain facilitates interaction with other transcriptional regulators in a manner that is highly dependent on the cellular microenvironment. Although widely expressed in the brain, its functional roles in the central nervous system have been largely uncharacterized. Our work extends previous reports of ZBTB7A’s importance in immune regulation by demonstrating its critical roles in cellular stress response phenotypes and neuropsychiatric disease. Previous studies using human IPSC-derived and mouse cell culture systems have identified ZBTB7A as a master regulator of astrocyte inflammatory transcription after LPS stimulation or neuronal injury. Using *in vivo* rodent models, we provide robust evidence that ZBTB7A functions as a glial epigenetic regulator that coordinates transcriptional programs to affect astrocytic homeostatic regulation, astrocyte-neuron communication, and behavioral adaptations during stress. Notably, this work is in agreement with recent reports of ZBTB7A involvement in MDD epigenetic regulatory processes, including functional enrichment of ZBTB7A binding sites in MDD-linked SNPs from human GWAS studies^73^, and upstream regulation of key long non-coding RNAs that mediate sex-differences in MDD traits^74^.

### Defining ZBTB7A-mediated mechanisms of astrocyte dysfunction

Our data suggest that ZBTB7A modulates astrocytic cellular functions though an epigenetic priming mechanism that reconfigures chromatin accessibility patterns to allow for exaggerated transcriptional responses to subsequent stressful events. We found ZBTB7A influences both upregulation of inflammatory pathways and the downregulation of crucial astrocytic homeostatic functions, particularly those involved in glutamate clearance, synaptic organization, and ionic regulation—processes that are disrupted in rodent stress models and are conserved in human MDD. *Ex vivo* electrophysiology and *in vivo* microendoscopic calcium imaging revealed that ZBTB7A-mediated astrocyte changes significantly alter OFC neuronal function after an acute stress exposure, including enhanced post-synaptic responses, presynaptic vesicle dynamics, and increased neuronal activity during social interaction with an aggressive mouse. Critically, our chemogenetic silencing experiments provide evidence for a causal link between astrocyte-mediated dysregulation of OFC function and maladaptive stress responsivity.

Our results are consistent with evidence showing that when astrocytes enter a “reactive” state––characterized by changes in gene expression and cellular activity––they can profoundly influence the activity of nearby neurons^75^. Astrocytes alter neuronal excitability and firing properties through glutamate uptake, potassium (K+) buffering, gliotransmission (e.g., adenosine or serine), and regulation of GABA signaling^75,76^ . During stress, these functions are disrupted, promoting increased neuronal excitability that may precipitate functional imaging and behavioral phenotypes observed in human MDD patients. Our results align with this model: we observed reduced expression and chromatin accessibility of the astrocytic glutamate transporter *Slc1a2* in CSDS OFC, which was reversed by Zbt-KD, providing a potential epigenetic mechanism connecting astrocytic dysfunction and aberrant neuronal activity during stress.

Interestingly, both astrocytic ZBT-OE SSDS and Zbt-KD CSDS altered presynaptic mechanisms of neuronal transmission, yet had opposite effects on stress-related behaviors. We posit that the decreased PPR in Zbt-KD + CSDS mice may reflect homeostatic compensatory mechanisms rather than behavaviroal drivers. While the precise physiological mechanisms through which ZBTB7A influences alterations in astrocyte-neuron communication to precipitate stress vulnerability remain unclear, our results provide ample evidence indicating that this transcription factor plays critical roles in the regulation of astrocytic homeostasis, astrocyte-neuron communication, and behavioral adaptations to chronic stress.

### Limitations and future directions

While our study provides strong evidence for a causal role of astrocytic ZBTB7A in stress vulnerability, several questions remain for future investigation. First, the precise mechanisms through which ZBTB7A reshapes chromatin and recruits co-regulators in astrocytes warrant further exploration, and may reveal additional points of stress-induced modulation. Second, although our study focuses on astrocytes, it is likely that ZBTB7A plays additional cell-type specific roles to coordinate epigenetic-mediated stress responses. Therefore, a more comprehensive understanding of how ZBTB7A and other chromatin regulators contribute to diverse cell-cell interactions amongst glia and neuronal subtypes during/following stress exposures will be important to understanding this process – particularly how potential changes in ZBTB7A-induced changes in glutamate uptake kinetics influence astrocyte-neuron communication during stress. Finally, given the higher prevalence of MDD in females, the question of sex differences in ZBTB7A expression or function will be extremely important for future studies. Here, we used only male mice for behavioral studies using the social defeat model, however, there remains much to be explored regarding how ZBTB7A, which has well characterized interactions with androgen receptors and sex hormone signaling pathways, may influence sex-dependent differences in stress response phenotypes. Further defining biomarkers of ZBTB7A activity and/or its downstream effectors will inform on its potential as a diagnostic indicator or predictor of treatment response in MDD patients. In summary, our cross-species findings support a critical role for astrocyte plasticity in the pathophysiology of MDD and stress-related disorders, and highlight the value of using epigenomic approaches to identify regulatory mechanisms underlying complex psychiatric disorders.

## Resource Availability

### Lead Contact

Requests for further information and resources should be directed to and will be fulfilled by the lead contact, Ian Maze (ian.maze@mssm.edu).

### Materials availability

This study did not generate new unique reagents, all materials are listed in the STAR resources table.

### Data and Code availability

All data, including raw (FASTQ files) and processed ATAC-seq data (OCRs, and raw / normalized count matrices) have been deposited in Gene Expression Omnibus, (GSE149871). Browsable UCSC genome browser tracks of processed data are available at: https://labs.icahn.mssm.edu/roussos-lab/mdd_atacseq. Raw and processed RNA-seq data and mouse astrocyte-specific ATAC-seq data is accessible through GEO Series accession number GSE214922 (RNA-seq) and GSE217568 (ATAC-seq). External validation sets: RNA-seq of MDD case/control postmortem human brains (GSE102556), TRAP-seq of astrocyte specific CSDS (GSE139684). No original code was used for this study. Any additional information required to reanalyze the data reported in this paper is available from the lead contact upon request.

## STAR Methods

### EXPERIMENTAL MODEL AND STUDY PARTICIPANT DETAILS

#### Human postmortem samples

Postmortem human orbitofrontal cortex (OFC, Brodmann Area 11) tissues were obtained from the Human Brain Collection at University of Texas Southwestern Medical Center (UTSW) under IRB approval. The cohort included 39 Caucasian individuals (20 MDD cases, 19 controls), matched for age, postmortem interval, and RNA integrity number. Tissue preservation was achieved as previously described^77^. Brains were placed on wet ice and transported to the UTSW Brain Bank facilities. Tissues were sliced, flash frozen in 2-methylbutane at −40 ^o^C, and stored at −80 °C. For each subject, the cause of death was determined by the Coroner Office, and toxicological screens were performed to obtain information on medication and illicit substance use at their time of death. The MDD group consisted of 20 (9 male and 11 female) individuals who met the Structured Clinical Interview for DSM-V (Diagnostic and Statistical Manual of Mental Disorders-V) Axis I Disorders: Clinician Version (SCID-I) criteria for Major Depressive Disorder. The control group comprised 19 subjects (12 male and 7 female Caucasians) with no history of MDD. Groups were matched for age, post-mortem interval and RNA integrity number (RIN). For all subjects, psychological autopsies were performed, giving us access to detailed information on psychiatric and medical histories, as well as other relevant clinical and sociodemographic data (see Table S1). Sex was explored and included as a co-variate in human transcriptomic/chromatin accessibility datasets. See Figure S1 and methods section for contribution of sex to variance in ATAC-seq data.

#### Mice

All animal procedures were conducted in accordance with NIH guidelines and approved by IACUC at the Icahn School of Medicine at Mount Sinai and Columbia University Zuckerman Institute. All mice used in the study were male. C57BL/6J mice were purchased from The Jackson Laboratory (RRID: IMSR_JAX:000664) between the ages of 6 and 12 weeks old. Retired breeder CD-1 mice were purchased from Charles River (RRID:IMSR_CRL:022). Mice were housed on a 12h light/dark cycle with *ad libitum* food and water access, except where specified for reward learning training.

#### Primary Human Astrocytes

Primary human astrocytes (PHAs) were obtained from Sciencell (#1800), derived from human cerebral cortex and frozen at first passage. Cells were maintained in Astrocyte medium (Sciencell #1801) on Matrigel-coated plates. Sex of the donor was not reported by the supplier.

#### Primary Mouse Astrocytes

Primary astrocytes were cultured from frontal cortical dissections of P1 mouse pups (C57BL/6J). Cortices were dissociated and plated in DMEM supplemented with 10% FBS and 1% penicillin-streptomycin. On DIV1, floating cells and neurons were removed by media change. Astrocytes were grown to confluency and subsequently used for downstream molecular assays. Sex of the donors was not determined.

### METHOD DETAILS

#### RNA Extraction

For human postmortem OFC, ~25mg of pre-sectioned flash-frozen tissue was utilized for RNA extraction. For mouse studies, animals were euthanized, and brains were removed whole and flash frozen (for bulk sequencing), or processed fresh for cell-type specific isolation with magnetically activated cell sorting (MACs). Dissected mouse brains were sectioned at 100 µm on a cryostat (bulk) or brain block (MACs), and GFP was illuminated using a NIGHTSEA BlueStar flashlight to micro dissect virally infected tissues with a 2mm punch. For both human and mouse experiments, OFC tissues were homogenized in Trizol (Thermo Fisher #15596026), and RNA was isolated on RNeasy Minelute Microcolumns (Qiagen #74204) following manufacturer’s instructions.

#### Bulk RNA-Sequencing

Samples were enriched for mRNA via polyA tail selection beads, and mRNA libraries were prepared using the Illumina Truseq RNA Library Prep Kit V2 (#RS-122–2001). Libraries were pooled and sequenced on the Illumina Novaseq platform, with an average read count of approximately 20 million paired end reads per sample.

RNA-seq data was pre-processed and analyzed as previously described^78^. Briefly, FastQC^79^ (Version 0.72) was performed on the concatenated replicate raw sequencing reads from each library to ensure minimal PCR duplication and sequencing quality. Reads were aligned to the hg38 or mouse mm10 genome using HISAT2^80^ (Version 2.1.0) and annotated against Ensembl v90. Multiple-aligned reads were removed, and remaining transcript reads were counted using featurecounts (Version 2.0.1). For mouse RNA-sequencing experiments with multiple groups, RUVg^81^ was performed to normalize read counts based on empirically determined control genes that do not vary between control and stress groups (i.e. genes with p-val > 0.5 based on a first-pass differential expression analysis performed prior to RUVg normalization). For human RNA-seq and mouse RNA-seq experiments with two groups, RUVr^81^ was performed to normalized read counts based on the residuals from a first-pass GLM regression of the unnormalized counts on the covariates of interest. The number of factors of variation, or RUV *k*, for each experiment is listed in Table S5). DESEQ2^82^ (Version 2.11.40.6) was used to perform pairwise differential expression analyses between indicated comparisons. Differentially expressed (DE) genes (listed in Table S5 for each experiment) were defined at FDR<0.1.

Threshold free Rank-Rank Hypergeometric Overlap (RRHO) heatmaps were generated to visualize transcriptome-wide gene expression concordance patterns as previously described^52^, using RRHO2^52^ (Version 1.0). For RRHO comparing ATAC-seq vs. RNA-seq, signed log p-value from the RNA-seq DESEQ2 output was ranked for each transcript that was also associated with a differentially accessible peak in the ATAC-seq.

For the human MDD dataset, we used the WGCNA package (Version 1.71)^26^ to construct the co-expression network for the top 2000 most variable genes in the set. We chose a suitable soft threshold power of 7 for scale-free network construction with the function pickSoftThreshold. The resulting gene co-expression network was visualized as the heatmap based on dissimilarity of TOM with hierarchical clustering dendrogram, and the number of genes in each module was counted. The correlation between modules and the trait of MDD was assessed by the Pearson correlation coefficients, with students *t*-test, and a *p* value of < 0.05 was considered statistically signidepificant. Gene ontology (GO) enrichment analysis was performed for genes in each significant module (and for GO analyses on DE genes from other experiments) with gprofiler^83^ (v0.2.3) (GO), idep (v2.0.1)^84^ (TRANSFAC/JASPAR databases) with total detected genes as background, and enrichR^85^ (for cell-type and human disease databases) to test for overrepresented gene categories in our list of DE genes. FDR for representative GO terms from the top 10 terms is calculated based on nominal P-value from the hypergeometric test. Gene Set Enrichment Analysis was performed using the ClusterProfiler^86^ package (Version 4.6.0) against GO to calculate gene set enrichment scores, and gene sets were ranked by adj. p-value^87^. Odds Ratio analyses were carried out on DE gene lists using the *GeneOverlap* R package version 1.26.0^88^.

##### FAN-sorting of neuronal and non-neuronal nuclei

50mg of frozen brain tissue was homogenized in cold lysis buffer (0.32M Sucrose, 5 mM CaCl2, 3 mM Mg(Ace)2, 0.1 mM, EDTA, 10mM Tris-HCl, pH8, 1 mM DTT, 0.1% Triton X-100) and filtered through a 40µm cell strainer. The flow-through was underlaid with sucrose solution (1.8 M Sucrose, 3 mM Mg(Ace)2, 1 mM DTT, 10 mM Tris-HCl, pH8) and subjected to ultracentrifugation at 24,000 rpm for 1 hour at 4°C. Pellets were thoroughly resuspended in 500µl DPBS and incubated in BSA (final concentration 0.1%) and anti-NeuN antibody (1:1000, Alexa488 conjugated, Millipore) under rotation for 1 hour, at 4 °C, in the dark. Prior to FANS sorting, DAPI (Thermoscientific) was added to a final concentration of 1µg/ml. DAPI positive neuronal (NeuN+), and non-neuronal (NeuN-) nuclei were sorted into tubes pre-coated with 5%BSA using a BD-FACSAria flow cytometer (BD Biosciences) equipped with a 70μm nozzle (Figure S6). 39 tissue dissections from 1 brain region were subjected to FANS, resulting in 78 (39 NeuN- and 39 NeuN+) distinct nuclear populations.

##### Generation of human ATAC-seq libraries

ATAC-seq reactions were performed using an established protocol^89^ with minor modifications. Following FANS, 50,000 sorted nuclei were centrifuged at 500 ×g for 10 min, 4°C. Pellets were resuspended in transposase reaction mix (25 μL 2x TD Buffer (Illumina #FC-121–1030) 2.5 μL Tn5 Transposase (Illumina #FC-121–1030) and 22.5 μL Nuclease Free H2O) on ice. Samples were incubated at 37°C for 30 min and then purified using the MinElute Reaction Cleanup kit (Qiagen #28204) according to the manufacturer’s instructions. Following purification, library fragments were amplified using the Nextera index kit (Illumina #FC-121–1011), under the following cycling conditions: 72°C for 5 minutes, 98°C for 30 seconds, followed by thermocycling at 98°C for 10 seconds, 63°C for 30 seconds, and 72°C for 1 minute for a total of 5 cycles. To prevent saturation due to over-amplification, a 5µl aliquot was then removed and subjected to qPCR for 20 cycles to calculate the optimal number of cycles needed for the remaining 45 μL reaction. The additional number of cycles was determined as follows: (1) Plot linear Rn vs. Cycle (2) Calculate the # of cycles that corresponds to 1⁄4 of maximum fluorescent intensity. In general, we found adding 4–6 cycles to this estimate yielded optimal ATAC-seq libraries, as determined by analysis on Bioanalyzer High Sensitivity DNA Chips (Agilent technologies #5067–4626). Libraries were amplified for a total of 13–19 cycles. Following PCR, ATAC-seq libraries were resolved on 2% agarose gels and fragments ranging in size from 100bp-1Kbp were excised and purified (Qiagen Minelute Gel Extraction Kit – Qiagen #28604). Libraries were quantified by quantitative PCR (KAPA Biosystems #KK4873) prior to sequencing. Libraries were sequenced on Hi-Seq2500 (Illumina) obtaining 2×50 paired-end reads. After quality controls (see below), 70 ATAC-seq libraries were retained for downstream analysis.

##### Human ATAC-seq Data Preprocessing

Raw sequencing reads were generated by the sequencing center demuxed and with adaptors trimmed. Reads were aligned to the hg19 reference genome with the STAR aligner (v2.5.0)^90^. Having a coordinate-sorted BAM, we further excluded reads that: (1) were mapped to more than one locus using samtools^91^; (2) were duplicated using PICARD (v2.2.4; http://broadinstitute.github.io/picard); and (3) mapped to the mitochondrial genome. Genotypes were called by GATK (v3.5.0)^92^. We performed: (1) indel-realignment; (2) base score recalibration; and (3) joint genotype calling across all samples for variants having a phred-scaled confidence threshold ≥ 10. We excluded clustered variants, variants in ENCODE blacklisted regions^93^, and variants not present in dbSNP v146^94^. Genotype concordance between samples was assessed using both the kinship coefficient calculated by KING v1.9^95^ and the fraction of concordant genotype calls. For these analyses, we kept only variants with minor allele frequencies (MAF) ≥ 25%. The two approaches yielded comparable results, with both indicating a clear and unambiguous separation of samples, and confirmed that neuronal and non-neuronal libraries originating from the same subject showed high genotype concordance scores (Figure S1M). The sex of the samples was assessed using three metrics: (1) the heterozygosity rate of chromosome X genotype calls outside the pseudoautosomal regions. For this, we removed variants with MAF < 5%. A high heterozygosity rate can indicate contamination in male samples. (2) The read counts of OCRs adjacent to *FIRRE* and *XIST* genes that are predominantly expressed in females. (3) Read counts in OCRs on chromosome Y outside the pseudoautosomal region. Using this approach, we detected and excluded two samples that were supposed to originate from a male subject, but they were genetically females. After their removal, all remaining samples matched the expected sex characteristics (Figure S1L).

For each sample, we calculated the following metrics: (1) total number of initial reads; (2) number of uniquely mapped reads; (3) fraction of reads that were uniquely mapped and additional metrics from the STAR aligner; (4) Picard duplication and insert metrics; (5) rate of reads mapped to the mitochondrial genome; (6) PCR bottleneck coefficient (PBC), which is an approximate measure of library complexity estimated as (non-redundant, uniquely mapped reads)/(uniquely mapped reads); (7) normalized strand cross-correlation coefficient (NSC) and relative strand cross-correlation coefficient (RSC), which are metrics that use cross-correlation of stranded read density profiles to measure enrichment independently of peak calling; (8) fraction of reads in peaks (FRiP), which is the fraction of reads that fall in detected peaks (see below for peak calling) and similarly the fraction of reads in only blacklisted peaks and the ratio between these two metrics. Table S2 describes the main QC metrics. On average, we obtained more than 27 million uniquely mapped paired end reads per sample. The rate of reads that mapped to the mitochondrial genome was below 2% since we generated ATAC-seq libraries using FANS separated nuclei, instead of whole cells. The bigWig tracks for each sample were manually inspected. A total of six libraries were excluded, having failed QC (including sex check) and/or visual inspection in IGV, leaving 70 libraries that were subjected to further analysis (Table S2).

#### Human ATAC-seq peak calling and read quantification

BAM-files of samples of the same diagnosis and cell type were merged and subsampled to a uniform depth of, at most, 454 million paired-end reads. We created a joint set of peaks requiring each peak to be called in at least one of the merged BAM-files. Peaks for OCRs were called by MACS (v2.1)^96^, using the following parameters^97^: *--keep-dup all -- shift −100 --extsize 200 --nomodel.* After removing peaks overlapping the blacklisted genomic regions, 371,820 peaks remained. Next, we counted how many reads for each sample overlapped consensus peaks using the featureCounts function in RSubread^98^ (v.1.15.0). We counted fragments (defined from paired end reads), instead of individual reads. This resulted in a sample by peak matrix of read counts.

We performed a statistical analysis of chromatin accessibility to detect genomic regions with significant differences in chromatin structure among neuronal and non-neuronal cells. First, we used the sample-by-peak read count matrix (70 samples by 371,820 OCRs). We subsequently excluded 1,178 OCRs using a criteria of “CPM ≥ 1 in at least 10% of the samples”, resulting in our final sample-by-peak read count matrix (70 samples by 370,642 OCRs). From here, we applied the trimmed mean of M-values (TMM)^99^ to normalize the read count followed by quantile normalization to achieve a balanced distribution of reads across samples of the same cell type.

Covariate exploration: Next, we tested whether we could find biological or technical sample-level covariates that affect the observed read count. For these covariates (e.g. number of peaks called in the sample, FRiP, chrM metrics, RSC and NSC, and Picard insert metrics), we normalized to the median of the cell. All 63 covariates were then tested for inclusion in differential analysis as detailed in the following: As a starting point for building the model to explain chromatin accessibility in the peaks, we selected cell type by diagnosis (2×2=4 levels) and sex (2 levels). To select additional covariates, we sought a good “average model” of chromatin accessibility over all OCRs. For each additional tested covariate, we asked how many OCRs showed an improved Bayesian Information Criterion (BIC) score minus how many showed a worse BIC score when the covariate was added to the” base” linear regression model. Here, we required that at least 5% of the OCRs showed a change of 4 in the BIC score, corresponding to “positive” evidence against the null hypothesis^100^. However, no covariate satisfied the BIC score criteria for inclusion. We were unable to find any covariate even after adjusting the threshold of minimal BIC (tested values = *2, 4, 10*) and/or minimal fraction of OCRs exceeding this threshold (tested values = *2%, 5%*). Overall, our final model included 2 variables (cell type by diagnosis and sex), where the number of levels for factor variables is noted here in square brackets. This model accounted for 5 DF.

For differential analysis, we used the *voomWithQualityWeights* function from the *limma* package^101^ to model the normalized read counts. Then, we performed differential chromatin accessibility analysis by fitting weighted least-squares linear regression models for the effect of each variable on the right-hand side on accessibility of each OCR:

chromatinaccessibility~celltype:diagnosis+Sex+(1\Person_ID)

To validate the relevance of differential OCRs, we applied the following strategies: permutation test and machine-learning test. For the former one, we randomly permuted MDD case/control status (*n* = 100 permuted datasets) and performed differential analysis using the same setting as for primary analysis. We measured (i) whether the sets of differential OCRs on permuted datasets are smaller compared to non-permuted datasets and (ii) whether the *P*-value rankings of differential OCRs on non-permuted datasets are close to normal distribution. For machine learning validation, we trained six machine learning models for prediction of MDD case/control status built on the reported set of (i) differential OCRs and (ii) the same number of randomly selected OCRs. We applied the repeated 5-fold cross-validation (krepeat = 10) and, additionally, we repeated the whole process 10 times with different sets of randomly selected OCRs. Then, we measured an improvement of prediction performance of the classifier based on differential OCRs over classifiers utilizing random OCRs. The following machine learning methods were tested, using the default setting in R-package caret^102^: Naive Bayes (nb), Random forest (rf), Nearest neighbor (knn), Logistic regression (multinom), SVM with linear kernel (svmLinear), and SVM with polynomial kernel (svmPoly).

We determined the genomic context per each OCR based on its proximity to the closest gene as assigned by ChIPSeeker^103^. For this, we created a transcript database using GenomicFeatures^104^ and Ensembl genes. The genomic context was defined as promoter (+/− 3kb of any TSS), 5’-UTR, 3’-UTR, exon, intron, and distal intergenic. We used GREAT approach^31^ to assign OCRs to genes and perform enrichment analysis with combined set of Gene Ontology^105^, biological processes with the curated canonical pathways from REACTOME^106^, KEGG^107^, and PID^108^, all accessed from MSigDB 6.0^109^. We further pruned highly similar gene sets by iteratively removing those with a Jaccard index ≥ 0.5, preferentially keeping the bigger gene set. This resulted in 4,590 gene sets (biological processes and pathways).

##### Overlap of OCRs with common variants in MDD

To determine whether the sets of neuronal, non-neuronal, and consensual OCRs as well as differential OCRs are enriched for common MDD GWAS variants^29^, we calculated partitioned heritability using LD-sc^28^. This analysis assesses if common genetic variants in the genomic regions of interest explain more of the heritability for a given trait than genetic variants not overlapping the genomic regions of interest, normalized by the number of variants in either category. The algorithm allows for correction of the general genetic context of the annotation using a baseline model of broad genomic annotations (like coding, intronic, and conserved regions). By using this baseline model, the algorithm focuses on enrichments above those expected from the general genetic context of the interrogated regions. We excluded the broad MHC-region (chr6:25–35MB) and, otherwise, used default parameters.

#### Transcription Factor Motif Matching

In order to identify candidates for DNA-binding proteins with recognition motifs enriched in our MDD-specific OCR set, we utilized the RSAT suite *peak-motifs,* a computational pipeline that discovers motifs in input sequences, and compares them with position-specific scoring matrix (PSSM) transcription factor databases^110,111^. Input sequences are scanned to predict binding sites, and the background model is a Markov chain of order 2 trained on the input sequences. Using *peak-motifs*, word-based analysis was first performed on the MDD-specific OCR set (n=183 sequences) with hexanucleotides (k *= 6*) and heptanucleotides (k *= 7*). The tool combines four pattern-discovery algorithms that utilize overrepresentation and positional bias as two criteria to detect significant oligonucleotide, which are then used as seeds to build probabilistic description of motifs (PSSMs), indicating residue variability at each position of the motif. Discovered motifs were compared with the JASPAR nonredundant core database of known transcription factor binding motifs to predict associated transcription factors (using *compare-matrices*). Several metrics are computed to measure the similarity between each matrix pair (including Pearson correlation, width normalized correlation). These metrics are converted to ranks, and a mean rank is computed to enable comparison between candidate factors. The *peak-motifs* pipeline discovered a motif (Figure 2A) that was significantly enriched in MDD-specific OCR sequences. The distribution of this motif within OCR sequences is shown in Figure 2A, indicating a relatively higher number of sites near sequence centers. For the top 5 candidate transcription factors identified as matches to this motif, the bar graph in Figure S2A displays the consensus score from the Human Protein Atlas ^112^ for expression in human brain for each factor. The mRNA expression data is derived from deep sequencing of RNA (RNA-seq) from 37 different normal tissue types.

In order to characterize the functional role for the discovered motif from the *peak-motifs* pipeline, we utilized *GOMo* (v5.3.3), from the MEME-suite of tools^38^ (Figure 2B). This approach calculates associations between a user-specified DNA regulatory motif [expressed as a position weight matrix (PWM)] and Gene Ontology (GO) terms, by computing an association score between the (putative) targets of the input TF motif and each GO term in the GO map. An empirically generated p-value for the enrichment of the GO term is also computed for the association score for each GO term with respect to the motif, based on the rank sum test null model.

##### Footprinting analysis

To determine the bound/unbound status of transcription factors in neuronal and non-neuronal cells as well as in MDD cases and controls, we performed footprinting analysis using TOBIAS (v. 0.12.4)^42^. Following the settings from our previous study^113^, we searched for the presence of 431 motifs representing 798 transcription factors (some motifs are shared due to their high similarity) in consensus OCRs of four merged BAM files representing both cell types & MDD diagnosis status. First, we ran the TOBIAS module ATACorrect to correct for Tn5 insertion bias in input BAM files, followed by TOBIAS ScoreBigwig to calculate footprinting scores across OCRs. Then, TOBIAS BINDetect combined footprinting scores with the information of transcription factor binding motifs to evaluate the individual binding positions of each transcription factor and determine whether a given position was bound by a given transcription factor or not for each condition, i.e. cell type and brain region. Finally, TOBIAS PlotAggregate was used to visually compare the aggregated footprints for select motifs.

#### QPCR

To measure mRNA gene expression for gene targets of interest, FAN-sorted nuclei (Figure S1R) from human postmortem OFC tissues were prepared as described above, with addition of RNAse inhibitor in the sorting collection buffer and pelleted for RNA extraction. Cultured human primary astrocytes (HPA) were washed with sterile PBS, scraped and pelleted for RNA extraction. For both nuclei samples and HPA cell samples, pellets were resuspended in RLT lysis buffer with 10% B-mercaptoethanol (B-ME), homogenized with a 22g needle and syringe, combined with equal volume 70% ethanol, and applied to Qiagen micro minelute column. RNA was washed, treated with DNAase, and eluted in 13ul of RNAse-free water, according to manufacturer’s instructions.

To measure *ZBTB7A* in bulk brain tissues, postmortem human OFC tissues were sectioned into 50 mg sections. Frozen mouse brains were sliced into 1mm coronal slices in a brain matrix, and 2mm OFC punches were removed. For both human and mouse tissues, sections were homogenized in Trizol (Thermo Fisher #15596026) with a motorized pestle, followed by chloroform extraction and precipitation with 70% ethanol. Samples were applied to a Qiagen micro minelute column, and RNA was washed, treated with DNAse, and eluted according to manufacturer’s instructions into 13ul RNAse-free water. For all RNA samples (derived from nuclei, cells, or brain tissues), 500ng of total RNA was utilized to synthesize cDNA using the Bio-Rad script cDNA synthesis kit (#1708891). From this reaction, 4 ng of cDNA was used to perform qPCR with PowerUp^™^ SYBR^™^ Green Master Mix (#A25742), according to the manufacturer’s instructions. Target gene CT values were averaged over 3 replicates, normalized to the reference gene (human brain – *HPRT1*, mouse brain – *Gapdh*), and the ΔΔCT was calculated. Graphs show experimental group fold change relative to controls, mean +/− SEM. Full list and sequences of primers used can be found in Supplementary Table S8.

#### Western Blotting

To measure protein expression, postmortem human OFC tissues were sectioned into 50 mg sections. Frozen mouse brains were sliced into 1mm coronal slices, and 2mm OFC punches were removed. For both human and mouse tissues, sections were homogenized in 200ml RIPA cell lysis buffer, 1X protease inhibitor cocktail and 1X phospho-stop inhibitor using a 1ml dounce homogenizer. Following homogenization, lysates were briefly sonicated with a probe sonicator for five 1s pulses. Protein concentrations were measured using the DC protein assay kit (BioRad), and 20 ug of protein was loaded onto 4–12% NuPage BisTris gels (Invitrogen) for electrophoresis. Proteins were then fast transferred to nitrocellulose membranes and blocked for 1 hr in 5% milk in PBS + 0.1% Tween 20 (PBST), followed by incubation with primary antibodies overnight at 4° C with rotation. The following antibodies were used: monoclonal rabbit anti-zbtb7a (Abcam #ab175918) (1:1000) for human blots, rabbit anti-Zbtb7a (Abcam #ab106592) (1:1000) for mouse blots, as well as rabbit anti-Gapdh (Abcam #ab9485) (1:10,000), and rabbit anti-H3.3 (Abcam #ab1791). After overnight primary antibody incubation, membranes were washed 3x in PBST (10 min) and incubated for 1 hr with horseradish peroxidase conjugated anti-rabbit (BioRad 170–6515, lot #64033820) secondary antibodies (1:10000; 1:50000 for anti-Gapdh antibody, BioRad) in 5% milk/PBST at RT. After three final washes with PBST, bands were detected using enhanced chemiluminescence (ECL; Millipore). Densitometry was used to quantify protein bands using ImageJ^114^ Software (NIH). Target protein measurements were normalized to Gapdh bands, and experimental group fold change was calculated relative to controls. Raw blots and indication of representative images utilized can be found in Figure S6.

#### Male Chronic Social Defeat Stress Paradigm

In order to investigate the expression of Zbtb7a in the context of a mouse model of stress, the Chronic Social Defeat Stress (CSDS) in males was performed as described previously ^45^. Briefly, a cohort of 20 8-week-old male C57BL/6J mice were randomly assigned to either the control or stress condition. Animals in the stress group underwent 10 consecutive days of a single 7-minute defeat session with an unfamiliar CD1 retired breeder male that had been previously screened for aggression towards C57BL/6J mice. Following the defeat session, the C57 mice spent 24 hours in the same cage as the CD1, separated by a perforated divider to allow for sensory contact. Control animals spent 24 hours in the same cage as a different male C57BL/6J for each day of the 10-day paradigm, separated by a perforated divider. 48 hours after the last defeat session, mice were assayed with the social interaction test.

#### Social Interaction Test

The Social Interaction (SI) test was performed as described previously^45,115^. Briefly, in the first trial, the subject mouse was allowed to freely explore an arena with an empty mesh cage inside an interaction zone. In the second trial, a CD1 target was put in the mesh cage, and the mouse was again allowed to explore the arena. Trials are recorded and scored by Ethovision software: SI ratio score was calculated as (time spent in interaction with target)/(time spent in interaction zone without target). Control mice typically have scores ≥ 1.0, indicating increased time spent investigating the unfamiliar mouse. In the stress mice, scores < 1.0 are defined as “avoidant” and mice are described as stress susceptible, while scores > 1.0 are defined as “non avoidant” and the mice are described as stress resilient. In a typical CSDS experiment, approximately 30% of WT mice will segregate into the stress resilient group^49^.

#### Viral Constructs

Viral vector constructs were generated as previously described^116^. Briefly, ZBTB7A overexpression plasmids (Origene Cat. #RC222759) were cloned into either a Lentiviral CMV-driven construct (Systems Biosciences Cat#CD516B-2) for use in cell culture experiments (shown in Figure S2) or a GFAP-GFP Adeno-associated virus (AAV) construct (Addgene plasmid #50473) for use in animal experiments (utilized in Figure 3–Figure 4). pAAV-GFAP-EGFP was a gift from Bryan Roth (Addgene plasmid # 50473; RRID:Addgene_50473). Lentiviral vectors contained either a ZBTB7A-HA tagged overexpression construct or an empty vector expressing RFP. AAV vectors contained either an ZBTB7A OE construct or GFP. For AAV vectors utilized in Figure 5, a miRNA targeting endogenous Zbtb7a was generated using the BLOCK-iT^™^ Pol II miR RNAi Expression Vector Kit with EmGFP (Thermo # K493600), in addition to a scramble negative control (Thermo Cat# K493600) (miR-neg) which forms a hairpin structure just as a regular pre-miRNA but does not target any known vertebrate gene. Constructs were packaged into GFAP driven AAV expression vectors to generate AAV-GFAP-Zbtb7a-miR-GFP and AAV-GFAP-mir-neg-GFP. Purified plasmids were sent to GENEWIZ for sequence validation. Plasmids were sent to Cyagen Biosciences for packaging into Lentivirus or AAV6 serotype viruses at high titer (>10^12 units).

Negative control sequence without 5’ overhangs:

GAAATGTACTGCGCGTGGAGACGTTTTGGCCACTGACTGACGTCTCCACGCAGTACATTT

Oligos used for Zbtb7a KD:

NM_010731.3_1062_top:

TGCTGTAGAAGTCCAAGCCATTGCAGGTTTTGGCCACTGACTGACCTGCAATGTTGGACTTCTA

NM_010731.3_1062_bottom:

CCTGTAGAAGTCCAACATTGCAGGTCAGTCAGTGGCCAAAACCTGCAATGGCTTGGACTTCTAC

#### Primary Human Astrocyte Cell Culture and LPS treatment

Primary Human Astrocytes isolated from human cerebral cortex and frozen at first passage were purchased from Sciencell (#1800) and cultured in Astrocyte medium (Sciencell #1801) on 50ug/ml coated Matrigel-coated plates (BD #354230). Cells were treated with lentivirus particles at MOI = ~2 to overexpress ZBTB7A or RFP. Approximately 72 hours after lentivirus transduction, PHAs were treated with 2ug puromycin to positively select for cells expressing each construct. After 6 days of selection, cells were collected for molecular analyses (Figure S2L–O). For testing ZBTB7A mRNA expression in inflammatory conditions, the cells were treated with either saline or LPS (Sigma Cat. # L2630) at 1 ug/ml for 8 hours and collected for molecular analyses (Figure S2P).

#### Primary Mouse Astrocytes

Primary astrocytes were cultured from frontal cortical dissections of mouse pups at P1, as previously described^117^. Briefly, cortices were dissociated, and diluted in 10% Fetal Bovine Serum (OmegaSci, FB-11)/1% penicillin-streptomycin in DMEM (Gibco, #11995–065) and plated at a density of one brain per uncoated T75 flask. On DIV 1, plates were tapped to dislodge neurons, and the media was changed to remove floating cells. Remaining astrocytes were maintained and grown to confluency and seeded at a density of approximately 3×106 cells/plate for subsequent experiments. Once confluent, the cells were treated with either saline or LPS (Sigma #L2630) and collected for molecular analyses (Figure S2Q).

#### TRAP-sequencing Data

Polyribosome immunoprecipitation was performed as described^118^. Briefly, mice were put through the CSDS paradigm, as described above, and ribosomes were homogenized with NP-40 (EMD Biosciences) and DHPC (Avanti Polar Lipids). Ribosomes were immunoprecipitated with anti-EGFP antibodies conjugated to Protein G magnetic Dynabeads (Invitrogen). The beads were washed, and RNA was extracted using Trizol and RNeasy columns (Qiagen). RNA was amplified using the Ovation RNA-seq System V2 (NuGEN). Libraries were sequenced on the Illumina HiSeq platform.

#### Immunohistochemistry

Mice were anesthetized with intraperitoneal (i.p.) injection of ketamine/xylazine (10/1 mg/kg) and then perfused transcardially with ice cold phosphate buffered saline (PBS) followed by ice cold 4% paraformaldehyde (PFA) in PBS. Next, brains were post-fixed in 4% PFA overnight at 4° C and then transferred into 30% sucrose in PBS for two days. Brains were then cut into serial 40 µm coronal slices in a cryostat at −20C. Free floating slices containing OFC were washed 3x in tris buffered saline (TBS), incubated for 30 min in 0.2% Triton-X in TBS to permeabilize tissue, and then incubated for 1 hr at RT in blocking buffer (0.3% Triton-X, 3% donkey serum in TBS). Brain slices were then incubated overnight on an orbital rotator at 4 degrees C with primary antibodies. 24 hours later, brain slices were washed 3x in TBS and then incubated for 2 hrs at room temperature (RT) with a fluorescent-tagged AlexaFluor 680 secondary antibody. Brain sections were then washed 3x in TBS, incubated with DAPI (1:10,000, lot # RK2297251, Thermo Scientific #62248) for 5 min at RT, mounted on Superfrost Plus slides (Fischer Scientific) and then coverslipped with Prolong Gold (Invitrogen #P36930). Immunofluorescence was visualized using a confocal microscope (Zeiss LSM 780 and W1 Yokogawa Spinning Disk). For quantification of Zbtb7a and GFP overlap with Gfap, images were split into respective color channels, and we calculated the Mander’s Correlation Coefficient^119^ using the *coloc2* package (version 2.0.2) on FIJI, which performs pixel intensity correlation and statistical testing. For GFP overlap we created an ROI to encompass Layer 5 OFC.

##### Antibodies used:

###### Primary

Chicken-anti-GFAP (astrocyte marker): Thermo Scientific # PA1–10004 (1:1000)

Mouse-anti-NeuN (neuronal marker): Millipore # MAB377 (1:1000)

Note that some of the viruses utilized express endogenous fluorescent markers: eGFP (Zbt-KD/GFP and ZBT-OE/GFP and Gcamp8f) and mCherry (Gi DREADD).

###### Secondary

Goat-anti-chicken Alexaflour 680: Thermo Scientific # A32934 (1:500)

Donkey-Anti-mouse Alexaflour 680: Thermo Scientific # A32788(1:500)

Donkey-anti-Rabbit Alexaflour 568: Abcam #ab175470 (1:500)

Goat-Anti-Armenian hamster: Jackson Immunoresearch #127-545-099 (1:500)

#### Stereotaxic Viral injection surgeries

Male C57BL/6J mice were anesthetized with a ketamine/xylazine solution (10/1 mg/kg) i.p., positioned in a stereotaxic frame (Kopf instruments) and 1 µl of viral construct was infused bilaterally into the OFC using the following coordinates: AP, 2.6 mm; ML, ±1.2 mm; V, 2.8 mm, angle 10°). Following surgery, mice received meloxicam (1 mg/kg) s.c. and topical antibiotic treatments for 3 days. All behavioral testing or electrophysiological recordings commenced 21 days after surgery to allow for maximal expression of the viral constructs. During collection of OFC tissues, OFC slices were examined for transgene expression in the correct location – subjects with off-target viral injections were removed from the study.

#### Subthreshold Social Defeat Paradigm

To investigate the role of Zbtb7a in stress vulnerability, we performed the Subthreshold variant of the Social Defeat Paradigm (SSDS) on a cohort of 8-week-old C57BL/6J male mice that were injected with either the rAAV6-GFAP-Zbtb7a OE construct or the rAAV6-GFAP-GFP empty control vector into the OFC 3 weeks previously. Half of each virus group was randomly assigned to the stress group or control group. The stress group underwent the SSDS paradigm as described previously^49^. Briefly, the stress mice were subjected to three 5-min defeat sessions with an aggressive CD1 male mouse consecutively on a single day, separated by a 15-minute rest period. The experimental mouse then spent 24 hours in the aggressor home cage, separated by a perforated divider to allow sensory exposure to the aggressor, and was then tested for social interaction as described above. Note that WT mice do not show behavioral deficits after the SSDS paradigm.

#### Magnetic Cell sorting

For magnetic-activated cell sorting, we collected virally transduced fresh OFC tissues, pooling 3 mice per *n*, and performed the MACs protocol, following manufacturer’s instructions. Briefly, OFC tissues were removed and washed in cold D-PBS, and tissue was dissociated using the Adult Brain Dissociation Kit, mouse and rat (Miltenyi # 130-107-677) enzyme kit in combination with the gentleMACs Octo Dissociator with Heaters (Miltenyi # 130-096-427). Samples were strained with MACs SmartStrainers (70uM, Miltenyi# 130-098-462), and spun at 300g for 10 minutes at 4C. Myelin debris was removed using myelin removal beads (Miltenyi # 130-096-733) in combination with the autoMACs Pro Separator. Samples were magnetically labeled with Anti-ACSA-2 Microbeads (Miltenyi #130-097-678) to isolate astrocytes with the autoMACs Pro Separator with the positive selection program. The negative fraction was subsequently incubated with Adult Non-neuronal Cell biotin-antibody cocktail (Miltenyi #130-126-603), followed by anti-biotin microbeads (Miltenyi #130-126-603) and then processed on the autoMACs Pro Separator to isolate neuronal cells via negative selection. For validation experiments in Figure S2D–G, negative fractions following astrocyte isolation were further processed with anti-Cd11b microbeads (Miltenyi #130-093-634) to isolate microglia, followed by incubation with anti-Pdgfra microbeads (Miltenyi # 130-094-543) to isolate immature oligodendrocytes, using the autoMACS Pro Separator prior to isolation of neuronal fraction, as described above. Isolated astrocyte and neuronal cells were then counted, with 50K cells separated for ATAC-seq, and the remainder of cells used for RNA extraction via trizol, followed by cleanup using the Qiagen Minelute kit. For the mouse ATAC-seq, MACs-isolated cells were processed according to the OMNI-ATAC protocol^120^, which has been optimized for fresh cells.

#### Mouse ATAC-seq Differential Accessibility Analysis

Raw sequencing reads were aligned to the mouse genome (mm10) using default settings of HISAT2^80^. Only uniquely mapped reads were retained. Alignments were filtered using SAMtoolsv1.19^121^ to remove duplicate reads. Peak calling was performed using MACSv2.1.124 with settings --nomodel --shift −100 --extsize 200. Peaks were filtered for FDR < 0.05. Differential analyses were performed using diffReps20 with a window size of 1 kb. A default p-value cutoff of 0.0001 was used. Peaks and differential sites were further annotated to nearby genes or intergenic regions using the region analysis tool from the diffReps package. DiffReps outputs can be found in Supplementary Table S6.

#### Reward Sensitivity Tasks and Instrumental Saccharin Behavior:

Animals were single housed and given restricted access to water (4h/day for 4d) before the start of the behavioral training. During the experiment, mice were given access to water for 2h each day (post-session). Reward-learning training was performed as previously described^122^, with minor modifications. The first stage of the experiment was four days of Pavlovian cue-reward association training for reinforcement with 0.2% saccharin-solution. Modular standard mouse operant chambers enclosed in light and sound blocking cubicles were used, equipped with white house lights and ventilation fans - interior dimensions: 55.69 × 38.1 × 40.64 cm; exterior dimensions: 63.5 × 43.18 × 44.45 cm; walls: 1.9 cm) (MedAssociates, Fairfax, VT). Each chamber contained two retractable levers and one central reward magazine containing a dipper calibrated to provide ~50ul of liquid saccharin reward per each reinforcement. Each daily session was 40-min (with operant levers retracted), in which mice learned to introduce their noses into the central reward magazine to get saccharin rewards, which were delivered every 60 s. A cue light above the magazine signaled reward delivery. Correct and incorrect saccharin retrieval was detected via infrared beam breaks upon head entry in the magazine and automatically recorded by MedPC software.

Next, mice were put through 5–7 days of 1 h sessions of operant learning training, in which mice were conditioned to lever press on a fixed-ratio 1 (FR1) schedule for *ad libitum* saccharin reinforcement, where one lever press = one reward. The basic settings were: session onset was indicated by illumination of the house light, and extension of both active and inactive levers; one active lever response (FR1) initiated magazine-cue light illumination and subsequent reward delivery, and following retrieval a 2.5 s inter-trial interval (ITI) was initiated; the session terminated after 1 hr.

In OE studies, after lever-press training as described above, mice were further trained on a reversal learning paradigm, using two levers positioned left and right of the central liquid reward magazine. For the baseline phase, mice went through 1 session/day for 8 days of training: On FR1, a response at the correct lever-initiated magazine light and reward delivery, the session terminated after 30 min. At the reversal phase, the previously incorrect lever was now correct and *vice versa*, so that non-reward-shift behavior was required; reversal testing lasted for an additional 8 days.

#### Forced Swim

The forced swim test (FST) was similarly conducted as previously described^49,123^. Mice were placed in a 4-liter glass beaker with 2L of room-temperature water for 7 minutes. Each session was recorded and scored by a blinded observer to record the number of seconds each mouse was immobile during the last 4 minutes of the test.

#### Singe-cell suspension preparation and Flow Cytometry

Single-cell suspensions from the brain tissue were prepared as described previously^124^. Briefly, virally transduced OFC tissue was dissected, minced and digested with 450 U/ml collagenase I, 125 U/ml collagenase XI, 60 U/ml DNase I and 60 U/ml hyaluronidase (Sigma) in PBS for 40 min at 37 °C. Samples were passed through a 70-μm cell strainer and mixed with 30% percoll layered on top of 70% percoll. The percoll gradient was centrifuged at 500 *g* for 30 min with the brake off. The cell fraction was collected and washed with PBS before downstream applications. Total viable cell numbers were quantified using counting beads (Thermo Fisher Scientific). Cell suspensions were stained with the antibody cocktail in PBS supplemented with 2% FBS and 0.5% BSA. The following monoclonal antibodies were used for flow cytometry analyses at a dilution of 1/700: anti-CD45 (BioLegend, clone 30-F11, 103147), anti-CD11b (BioLegend, clone M1/70, 101226), anti-CD11c (Biolegend, clone N418, 117333), anti-TREM2 (R&D Systems, clone 237920, FAB17291P), anti-P2RY12 (Biolegend, clone S16007D, 848003), anti-ASCA2 (Miltenyi Biotec, clone REA969, 130-116-245), anti-MHCII (BioLegend, clone M5/114.152, 107602) and anti-CCR2 (R&D systems, clone 475301, MAB55381). Viable cells were identified through negative staining for Zombie NIR (BioLegend). Data were acquired on a Cytek Aurora and analyzed with FlowJo (Tree Star). Flow cytometry gating strategy shown in Figure S6 included all cells, singlets, live cells and cell populations were identified as astrocytes (ACSA2^+^CD45^−^) or microglia (CD45^mid^P2RY12^+^CD11b^+^).

#### Local Field Potential Electrophysiology Recordings

Male C57BL/6J mice (age approximately 60 days) were deeply anesthetized with isoflurane and then decapitated, followed by rapid removal and chilling of the brain. Coronal slices (300 µm thick) were prepared using a Compresstome vibrating microtome (Precisionary, Natick, MA), in ice-cold sucrose cutting solution (in mM: 215 sucrose, 2.5 KCl, 1.6 Na_2_HPO_4_, 26 NaHCO_3_, 4 MgSO_4_, 1 CaCl_2_ and 20 glucose). The slices then were transferred to artificial cerebrospinal fluid (ACSF; in mM: 120 NaCl, 3.3 KCl, 1.2 NaHPO_4_, 26 NaHCO_3_, 1 MgSO_4_, 2 CaCl_2_ and 11 glucose; pH 7.2, 300 mOsM; bubbled with 95% O_2_/5% CO_2_) at 32°C for 30 minutes, after which they were transferred to room temperature ACSF and allowed to recover for at least one hour. Recordings were obtained in a submersion recording chamber superfused with ACSF (1 mL/min) at room temperature. A concentric bipolar stimulating electrode was placed in layer 1 of the orbital frontal cortex to evoke synaptic responses using a 100 µs stimulus delivered by an IsoFlex stimulus isolator (AMPI, Jerusalem, Israel). A glass Ag/AgCl electrode filled with ACSF recorded field excitatory synaptic potentials (fEPSPs) from layer 5. Recordings were acquired using Axoclamp 2A and Axopatch 1D amplifiers, Digidata 1440A analog-digital convertor, and pClamp software 10 (all from Molecular Devices, San Jose, CA). Signals were low pass filtered at 2 kHz and digitized at 10 kHz. An input-output (I-O) curve was constructed by recording fEPSPs in response to stimuli ranging from 100–800 µA (average of three fEPSPs per stimulus strength, recorded at intervals of 20 seconds between stimuli, starting with the lowest intensity). Given the proximity of the recording electrode to the stimulating electrode within respective layers of the OFC, we plotted peak amplitude (instead of peak slope), to avoid effects of recording artifacts. Following construction of an I-O curve, the stimulus intensity that evoked a fEPSP of ~50% of maximum amplitude was used in paired-pulse ratio (PPR) and rundown experiments. Separate slices from the same animals were used for the PPR and rundown experiments. To evaluate PPR, paired stimuli were delivered every 60 seconds with decreasing interstimulus intervals (100, 50, and 20 ms). Each interstimulus interval was repeated three times and the resulting potentials were averaged. The ratio of the average amplitude of fEPSP2/fEPSP1 was calculated. For rundown experiments, a single 30-s train was delivered at 10 Hz after establishment of a stable baseline. The percentage change in fEPSP amplitude from baseline was calculated. All data were graphed as means ± SEM.

#### Patch Clamp Electrophysiology Recordings

Mice were deeply anesthetized with isoflurane and decapitated. The brain was rapidly removed and chilled in cutting artificial CSF (ACSF) containing (in mM): N-methyl-D-glucamine 93, HCl 93, KCl 2.5, NaH_2_PO_4_ 1.2, NaHCO_3_ 30, HEPES 20, glucose 25, sodium ascorbate 5, thiourea 2, sodium pyruvate 3, MgSO_4_ 10, and CaCl_2_ 0.5, pH 7.4. The brain was embedded in 2% agarose and coronal slices (300 µm thick) were made using a Compresstome (Precisionary Instruments). Brain slices were allowed to recover at 31 ±1 °C in ACSF for 30 minutes and thereafter at room temperature in holding ACSF, containing (in mM): NaCl 92, KCl 2.5, NaH_2_PO_4_ 1.2, NaHCO_3_ 30, HEPES 20, glucose 25, sodium ascorbate 5, thiourea 2, sodium pyruvate 3, MgSO_4_ 2, and CaCl_2_ 2, pH 7.4. After at least 1 hour of recovery, the slices were transferred to a submersion recording chamber and continuously perfused (2–4 mL/min) with recording ACSF containing (in mM, unless indicated otherwise): NaCl 124, KCl 2.5, NaH_2_PO_4_ 1.2, NaHCO_3_ 24, HEPES 5, glucose 12.5, MgSO_4_ 2, CaCl_2_ 2, 10 µM CNQX, and 100 µM gabazine, pH 7.4. All solutions were continuously bubbled with 95% O_2_ / 5% CO_2_. Pyramidal neurons located in Layer 5 of OFC were visually identified with infrared differential contrast optics (BX51; Olympus). Whole-cell patch-clamp recordings were performed at room temperature using a Multiclamp 700A amplifier (Molecular Devices). For excitability experiments, pipettes (3–5 MΩ) pulled from borosilicate glass were filled with solution containing (in mM): K-gluconate 115, HEPES 10, KCl 20, MgATP 5, MgCl_2_ 1.5, Na2GTP 0.5, Na-phosphocreatine 10, and EGTA 2, pH 7.25. Cells were allowed to stabilize for 3 minutes after breakthrough in I=0 mode. Data acquisition (signals were low pass filtered at 10 kHz in I-clamp mode and at 3 kHz in V-clamp mode and digitized at 10 kHz) and analysis was performed with pClamp 11 software (Molecular Devices) and GraphPad Prizm 10 (GraphPad software). Action potentials were evoked in current-clamp mode in response to 1-s depolarizing steps (20 steps in 10 pA intervals).

For whole cell EPSP and paired pulse (PP) recordings of EPSCs, the pipette solution contained (in mM) Cs-gluconate 122, HEPES 10, KCl 5, MgATP 5, Na2GTP 0.5, QX314 1, and EGTA 1, pH 7.2. The electrode placements and procedure of stimulation were similar to fEPSP recordings mentioned above.

To study the decay kinetics EPSPs were recorded in current clamp (I=0) mode. Square pulses of varying intensities (0.2 ms long; 30–200 µA) were used to find the maximum amplitude response. After a delay of at least 60 seconds, 5 stimuli were delivered at a rate of 0.2 Hz. To calculate the decay time constant (tau), the 2^nd^, 3^rd^ and 4^th^ responses were averaged, and the decay was fitted to a single exponential using the Chebyshev method over a 1-s interval beginning with the EPSP peak.

For paired pulse EPSCs were recorded at VH = −80 mV. The procedure of finding the appropriate stimulation intensity for each cell was same as above. To evaluate PPR, paired stimuli were delivered every 60 seconds with increasing interstimulus intervals (10, 20, 50, and 100 ms). All data were graphed as means ± SEM.

#### Microendoscopy In vivo Calcium Imaging

Mice were injected bilaterally with 250nL of viral vector expressing a GCaMP (AAV9-syn-GCamp8f, Addgene Cat# 162376-AAV9) + 500nL of AAV-GFAP-ZBT-OE virus or AAV-GFAP-RFP to Layer 5 of OFC (AP, 2.6 mm; ML, ±1.2 mm; V, 2.4 mm from dura). After viral injection, a ProView Integrated Lens 1.0mm x 4.0mm with attached baseplate (Inscopix # 1050–004637) was implanted above the viral infusion site (AP, 2.6 mm; ML, ±1.2 mm; V, 2.3 mm from dura). The GRIN lens baseplate was secured to the skull using Metabond Dental Cement (Parkell C&B Metabond Quick Adhesive Cement System Cat# S380). Three weeks following the implantation surgery, calcium signal and field of view was validated, and baseline calcium transients were recorded while mice were in their home cage (10 minutes) with the Incopix nVista 3.0 Data Acquisition Box and IDAS Software v2.3. 48 hrs later we recorded synchronized behavior and calcium transients during pre-SSDS social interaction test with the nVista 3.0 and nVision 1.4. One week later, all mice were put through SSDS, followed by a second synchronized recording during the social interaction test 48 hours after the defeat session. After behavior recordings, mice were perfused and OFC slices were imaged to visualize lens placement by registering each slice to the scalable Allen brain atlas^125^ and marking the bottom end of the lens for each subject.

#### Pre-processing of microendoscopy calcium imaging data

Pre-processing was done with IDEAS Inscopix Data Processing Software (v 25.1.4) using the end-to-end CNMF-E pipeline (v 5.0.2). Calcium recordings were temporally down sampled to 10Hz and processed through a spatial bandpass filter with global mean subtraction to remove low spatial frequency content and out-of-focus cells. Motion artifacts were removed using an image registration method that estimates a translation that minimizes the difference between the transformed frame and the reference frame (global mean projection). We next applied a constrained non-negative matrix factorization (CNMF) algorithm to define the spatial location and temporal dynamics of individual neurons in each imaging recording resulting in cell footprints and fluorescence traces for each neuron. The change in fluorescence across the recording was calculated in units delta fluorescence (dF) as the average fluorescence activity of all pixels in a cell, scaled so that each spatial footprint has a magnitude of 1. Subsequently, the trace activity of each individual neuron was calculated as delta fluorescence over fluorescence noise (dF/F) by dividing each temporal trace with its respective estimated noise level.

#### Calculating neuron activity synchronized with social interaction behavior

To compare neural activity pre/post SSDS stress, we compared calcium recordings during social interaction tests taken pre and post SSDS from the same mice and pre-processed them as a time series to extract calcium traces from the same individual neurons registered across the two epochs. The average activity of each neuron was rescaled to have a mean of 0 and a standard deviation of 1, removing differences in baseline activity and scale, allowing comparison of the relative variance between cells on a common scale. Finally, the pairwise difference in rescaled mean neural activity during pre/post SSDS social interaction test was calculated and plotted for each neuron (Figure 4J and Figure S4E).

#### Identification of neurons significantly modulated by SI entry

To identify individual neurons that were significantly modulated by entry into the social interaction zone during social interaction testing, we first used Ethovision (v15) to collect timestamps of when each mouse entered and exited the social interaction zone from the synchronized nVision behavior recordings, defined as one body length around the plexiglass enclosure holding the unfamiliar aggressor CD-1 mouse. If the entry and exit time stamps were less than 2 seconds, these social interaction “bouts” were combined into a single bout. We applied the Inscopix Peri-event workflow pipeline (v5.7.0), to quantify how each neuronal signal varies before and after SI entry timestamps, over the time window of interest (1s to 3s post social zone entry). This tool computes the change in neural activity of both the entire population of neurons for each subject and each individual neuron, classifying them into up-modulated, down-modulated, or non-modulated sub-populations: the peri-event change in activity is calculated by taking the difference between the post-event and pre-event activity, and comparing it to a null distribution generated by randomly circularly permuting the event times relative to the neural time series (1000 shuffles). Cells are classified as up-modulated if the bootstrap probability is less than α/2 for a two-tailed test, down-modulated if the bootstrap probability is greater than 1-α/2, and non-modulated if the bootstrap probability is between α/2 and 1-α/2, where α = 0.05 (Figure 4K). After each individual cell has been classified, they are grouped into up-modulated, down-modulated, and non-modulated subpopulations, and the average activity of each subpopulation is plotted (example Figure S4H).

#### Calculation of mean neuronal activity while in social interaction zone

To calculate the mean activity of individual neurons while in the Social Interaction Zone, we used Ethovision (v15) to collect timestamps of when each mouse was inside the social interaction zone for the Pre-SSDS and Post-SSDS social interaction test recordings, defined as one body length around the plexiglass enclosure holding the unfamiliar aggressor CD-1 mouse. If the entry and exit time stamps were less than 2 seconds, these social interaction “bouts” were combined into a single bout. Using these entry and exit timestamps, we applied the Inscopix IDEAS tool Compare Neural Activity Across States (v1.1.0) which calculates the mean activity (dF/F) of individual neurons while in the social interaction zone for each subject.

#### Calculating inter-cell synchrony: Cell-cell activity correlation

To determine how synchronous the neurons were after CSDS, we used the Inscopix IDEAS tool Compare Neural Circuit Correlations Across States (v2.1.1) to calculate pairwise correlations between cell traces for each subject while in the social interaction zone.

#### Chemogenetic Manipulation

In order to determine if neuronal hyperexcitability contributes to the observed behavioral effects of ZBTB7A OE, we performed the Subthreshold variant of the Social Defeat Paradigm (SSDS) on a cohort of 8 week old C57BL/6J male mice that were injected with the pAAV-hSyn-hM4D(Gi)-mCherry to express the inhibitory Gi DREADD (Addgene #50475-AAV2), in combination with either the rAAV6-GFAP-Zbtb7a OE construct or the rAAV6-GFAP-GFP empty control vector into the OFC 3 weeks previously. Half of each virus group was randomly assigned to the ZBT OE or GFP viral group. Both viral groups underwent the SSDS paradigm as described previously^49^. The experimental mouse then spent 24 hours in the aggressor home cage, separated by a perforated divider to allow sensory exposure to the aggressor. The mice were then single housed for 24 hours, and then half of each viral group was injected with either the DREADD agonist Deschloroclozapine^69^ (Tocris # 7193) at 1ug/kg in 1% DMSO or vehicle. Fifteen to twenty minutes post-injection, the mice were tested for social interaction as described above.

### QUANTIFICATION AND STATISTICAL ANALYSIS

Statistical analyses were conducted using GraphPad Prism (v10). Normality was assessed prior to parametric testing. Group differences were analyzed using two-tailed t-tests or ANOVA followed by Sidak’s or Tukey’s post hoc multiple corrections test. All data is shown as mean ± SEM. Statistical significance was defined as p < 0.05. Sample sizes (n), test statistics, and exact p-values are reported in Table S7_stats.

## Supplementary Material

1

2 **Table S1_demo**: Demographics of the postmortem brain cohort. Related to Figure 1.

3 **Table S2_qc**: Quality control metrics of ATAC-seq dataset of postmortem brain cohort. Related to Figure 1 and Figure S1.

4 **Table S3_dac**: Differential analysis between neuronal and non-neuronal samples as well as among MDD cases and controls in ATAC-seq dataset of postmortem brain cohort. Related to Figure 1.

5 **Table S4_gsea**: Gene set enrichment analysis for cell type and disease-specific sets of OCRS. Related to Figure 1.

6 **Table S5_deseq2:** DESEQ2 outputs for all RNA-seq experiments. Related to Figure 3 and Figure 5.

7 **Table S6_diffreps:** Diffreps outputs for mouse ATAC-seq experiments. Related to Figure 3 and Figure 5.

8 **Table S7_stats**: Full statistical information for Extended Data Figures.

9 **Table S8_primers**: Full list and sequences of all primers used in qPCR experiments. Related to Figure 2 and Figure S2.

## Figures and Tables

**Table:** KEY RESOURCES TABLE

REAGENT or RESOURCE	SOURCE	IDENTIFIER
**Antibodies**		
Rabbit anti-ZBTB7A (human)	Abcam	Cat# ab175918; RRID: AB_3696416
Rabbit anti-ZBTB7A (mouse)	Abcam	Cat# ab106592; RRID: AB_10866676
Rabbit anti-GAPDH	Abcam	Cat# ab9485; RRID: AB_307275
Rabbit anti-H3.3	Abcam	Cat# ab1791; RRID: AB_302613
Goat Anti-Rabbit-HRP	Bio-Rad	Cat# 170-6515; RRID: AB_11125142
Chicken anti-GFAP	Thermo Scientific	Cat# PA1-10004; RRID: AB_1074620
Mouse anti-NeuN	Millipore	Cat# MAB377; RRID: AB_2298772
Goat anti-chicken Alexa Fluor 680	Thermo Scientific	Cat# A32934; RRID: AB_2762846
Donkey anti-mouse Alexa Fluor 680	Thermo Scientific	Cat# A32788; RRID: AB_2762831
Donkey anti-rabbit Alexa Fluor 568	Abcam	Cat# ab175470; RRID: AB_2783823
Goat anti-Armenian hamster	Jackson ImmunoResearch	Cat# 127-545-099; RRID: AB_2338996
Anti-CD45 (clone 30-F11)	BioLegend	Cat# 103147; RRID: AB_2564383
Anti-CD11b (clone M1/70)	BioLegend	Cat# 101226; RRID: AB_830642
Anti-CD11c (clone N418)	BioLegend	Cat# 117333; RRID: AB_11204262
Anti-TREM2 (clone 237920)	R&D Systems	Cat# FAB17291P; RRID: AB_884528
Anti-P2RY12 (clone S16007D)	BioLegend	Cat# 848003; RRID: AB_2721644
Anti-ACSA2 (clone REA969)	Miltenyi Biotec	Cat# 130-116-245; RRID: AB_2727423
Anti-MHCII (clone M5/114.15.2)	BioLegend	Cat# 107602; RRID: AB_313317
Anti-CCR2 (clone 475301)	R&D Systems	Cat# MAB55381; RRID: AB_3658462
**Bacterial and virus strains**		
AAV-GFAP-GFP	pAAV-GFAP-EGFP was a gift from Bryan Roth, generated as part of the Illuminating the Druggable Genome (IDG) program sponsored by the NIH Common Fund.	Addgene Cat# 50473
AAV9-syn-GCaMP8f	Zhang et. al., 2020	Addgene Cat# 162376-AAV9
pAAV-hSyn-hM4D(Gi)-mCherry	pAAV-hSyn-hM4D(Gi)-mCherry was a gift from Bryan Roth,generated as part of the Illuminating the Druggable Genome (IDG) program sponsored by the NIH Common Fund.	Addgene #50475-AAV2
Pokemon (ZBTB7A) (NM_015898) Human Tagged ORF Clone	Origene	Cat.#RC222759
pCDH-CMV-MCS-EF1α-RFP+Puro Cloning and Expression Lentivector	Systems Biosciences	Cat#CD516B-2
**Biological Samples**		
Postmortem human OFC (BA11) (Table S1)	Human Brain Collection, UTSW	N/A
**Chemicals, Peptides, and Recombinant Proteins**		
Deschloroclozapine	Tocris	Cat# 7193
**Critical Commercial Assays**		
Nextera Index Kit	Illumina	Cat# FC-121-1011
BLOCK-iT^™^ Pol II miR RNAi Expression Vector Kit with EmGFP	Thermofisher	Cat# K493600
Adult Brain Dissociation Kit	Miltenyi	Cat# 130-107-677
Anti-ACSA-2 Microbeads	Miltenyi	Cat# 130-097-678
Myelin Removal Beads	Miltenyi	Cat# 130-096-733
**Deposited data**		
Raw FASTQ and processed ATAC-seq data for human OFC samples	This study	GSE149871
Raw FASTQ and processed RNA-seq data for mouse samples	This study	GSE214922
Raw FASTQ and processed ATAC-seq data for mouse samples	This study	GSE217568
RNA-seq of MDD case/control postmortem human brains	Labonte et al., 2017	GSE102556
Astrocyte specific TRAP-seq data across three brain regions in CSDS mice	This study	GSE139684
**Experimental models: Cell Lines**		
Primary Human Astrocytes (Cortical)	Sciencell	Cat#1800
**Experimental models: Organisms/strains**		
Mouse: C57BL/6J	Jackson Laboratory	RRID: IMSR_JAX:000664
Mouse: CD-1 retired breeders	Charles River	RRID: IMSR_CRL:022
**Oligonucleotides**		
Negative control sequence without 5’ overhangs: GAAATGTACTGCGCGTGGAGACGTTTTGGCCACTGACTGACGTCTCCACGCAGTACATTT	ThermoFisher	Cat # K493600
Oligos used for Zbtb7a KD (top): NM_010731.3_1062_top: TGCTGTAGAAGTCCAAGCCATTGCAGGTTTTGGCCACTGACTGACCTGCAATGTTGGACTTCTA	This paper	N/A
Oligos used for Zbtb7a KD (bottom): NM_010731.3_1062_bottom: CCTGTAGAAGTCCAACATTGCAGGTCAGTCAGTGGCCAAAACCTGCAATGGCTTGGACTTCTAC	This paper	N/A
Full list of primers used for qPCR is in Table S8_primers	N/A	N/A
**Software and algorithms**		
FastQC v0.72	Andrews, 2010	https://www.bioinformatics.babraham.ac.uk/projects/fastqc/
HISAT2 v2.1.0	Kim et al., 2019	https://daehwankimlab.github.io/hisat2/
featureCounts v2.0.1	Liao et al., 2014	http://bioinf.wehi.edu.au/featureCounts/
DESeq2 v1.40.2	Love et al., 2014	https://bioconductor.org/packages/release/bioc/html/DESeq2.html
RRHO2 v1.0	Cahill et al., 2018	https://github.com/igordot/genomics/blob/master/rrho2.R
WGCNA v1.71	Langfelder and Horvath, 2008	https://horvath.genetics.ucla.edu/html/CoexpressionNetwork/Rpackages/WGCNA/
gProfiler	Reimand et al., 2016	https://biit.cs.ut.ee/gprofiler/
iDEP	Ge et al., 2018	http://bioinformatics.sdstate.edu/idep/
enrichR	Chen et al., 2013	https://maayanlab.cloud/Enrichr/
clusterProfiler v4.6.0	Yu et al., 2012	https://bioconductor.org/packages/release/bioc/html/clusterProfiler.html
GeneOverlap v1.26.0	Shen and Sinai, 2019	https://bioconductor.org/packages/release/bioc/html/GeneOverlap.html
STAR v2.5.0	Dobin et al., 2013	https://github.com/alexdobin/STAR
SAMtools v1.9	Li et al., 2009	http://www.htslib.org/
Picard v2.2.4	Broad Institute	http://broadinstitute.github.io/picard/
GATK v3.5.0	McKenna et al., 2010	https://gatk.broadinstitute.org/
KING v1.9	Manichaikul et al., 2010	https://www.kingrelatedness.com/
MACS2 v2.1	Zhang et al., 2008	https://github.com/macs3-project/MACS
Rsubread v1.15.0	Liao et al., 2013	https://bioconductor.org/packages/release/bioc/html/Rsubread.html
limma	Ritchie et al., 2015	https://bioconductor.org/packages/release/bioc/html/limma.html
caret	Kuhn, 2008	https://cran.r-project.org/web/packages/caret/index.html
ChIPseeker	Yu et al., 2015	https://bioconductor.org/packages/release/bioc/html/ChIPseeker.html
GenomicFeatures	Lawrence et al., 2013	https://bioconductor.org/packages/release/bioc/html/GenomicFeatures.html
GREAT	McLean et al., 2010	http://great.stanford.edu/public/html/
RSAT peak-motifs	Medina-Rivera et al., 2015	http://rsat.sb-roscoff.fr/
GOMo (MEME-suite v5.3.3)	Buske et al., 2010	https://meme-suite.org/meme/tools/gomo
TOBIAS v0.12.4	Bentsen et al., 2020	https://github.com/loosolab/TOBIAS
FIJI coloc2 v2.0.2	Schindelin et al., 2012	https://imagej.net/software/fiji/
Ethovision v15	Noldus	https://www.noldus.com/ethovision
Flowjo	BD Biosciences (previously Tree Star)	https://www.flowjo.com/
pCLAMP 10 (Clampfit)	Molecular Devices	https://www.moleculardevices.com/products/axon-patch-clamp-system
Prism v10	GraphPad	https://www.graphpad.com/
Inscopix IDPS v25.1.4	Inscopix	https://www.inscopix.com/products/software/
Inscopix IDEAS tools	Inscopix	https://www.inscopix.com/products/software/
**Other**		
ProView Integrated Lens 1.0mm x 4.0mm	Inscopix	Cat # 1050-004637
		

## Highlights

- MDD epigenomic risk converges on glia in the OFC, a key hub for clinical symptoms
- ZBTB7A, a TF identified in MDD, regulates stress-induced astrocytic transcription
- ZBTB7A-mediated astrocyte dysfunction drives stress-linked OFC neuronal hyperactivity
- Modulating ZBTB7A reverses chronic stress-induced molecular and behavioral deficits
