# Supplementary material for: Major Depressive Disorder associated dysregulation of ZBTB7A in orbitofrontal cortex promotes astrocyte-mediated stress susceptibility
Source: Neuron. Author manuscript; Available in PMC 2025 Jul 1. (PMC12210276; doi:10.1016/j.neuron.2025.05.023)
Supplement: 1 [file NIHMS2084664-supplement-1.pdf]

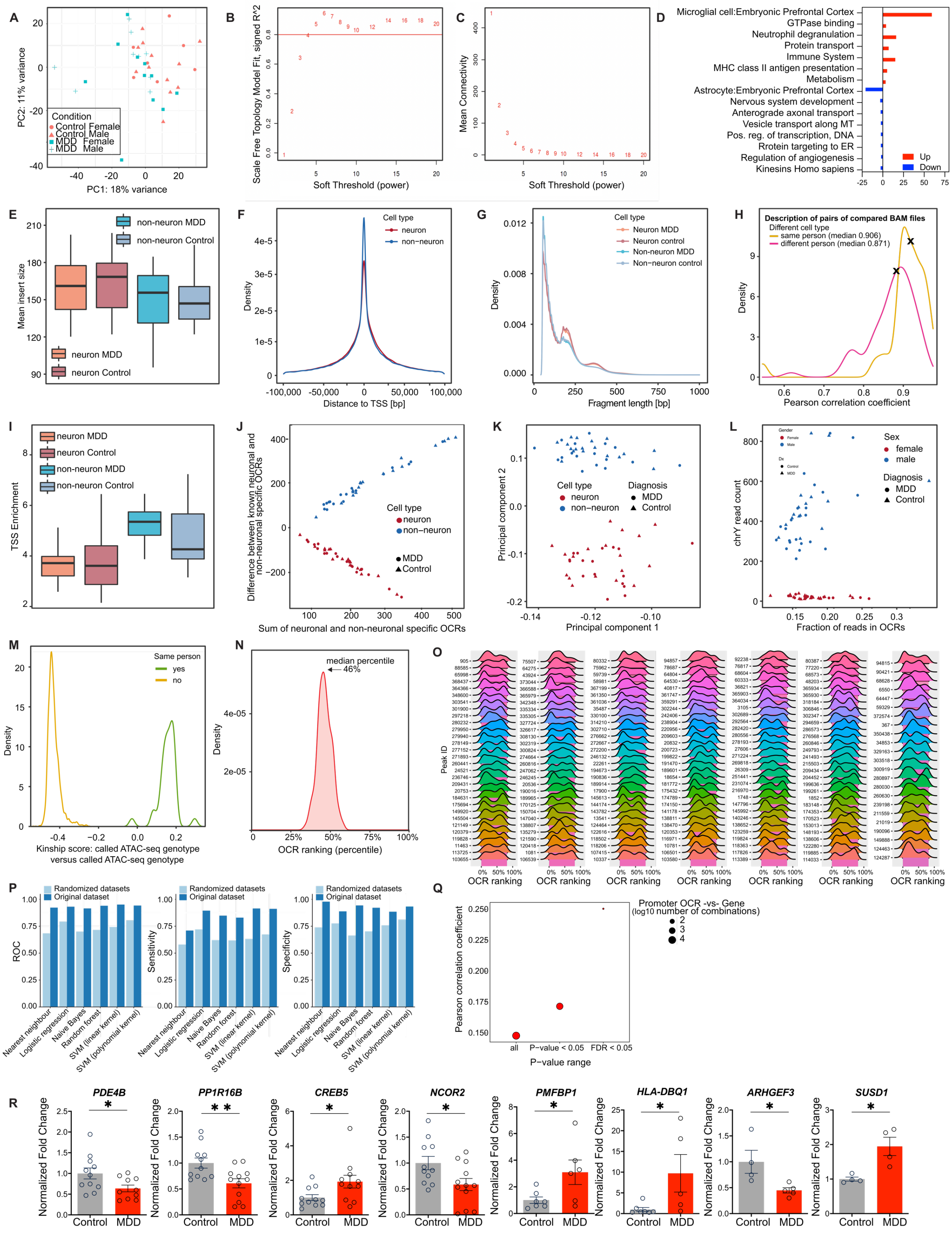
A
PC2: 11% variance
PC1: 18% variance
Condition
Control Female
Control Male
MDD Female
MDD Male
B
Scale Free Topology Model Fit, signed R^2
Soft Threshold (power)
C
Mean Connectivity
Soft Threshold (power)
D
Microglial cell:Embryonic Prefrontal Cortex
GTPase binding
Neutrophil degranulation
Protein transport
Immune System
MHC class II antigen presentation
Metabolism
Astrocyte:Embryonic Prefrontal Cortex
Nervous system development
Anterograde axonal transport
Vesicle transport along MT
Pos. reg. of transcription, DNA
Rotein targeting to ER
Regulation of angiogenesis
Kinesins Homo sapiens
Up
Down
E
non-neuron MDD
non-neuron Control
neuron MDD
neuron Control
Mean insert size
F
Cell type
neuron
non−neuron
Density
Distance to TSS [bp]
G
Cell type
Neuron MDD
Neuron control
Non-neuron MDD
Non−neuron control
Density
Fragment length [bp]
H
Description of pairs of compared BAM files
Different cell type
same person (median 0.906)
different person (median 0.871)
Density
Pearson correlation coefficient
I
neuron MDD
neuron Control
non-neuron MDD
non-neuron Control
TSS Enrichment
J
Difference between known neuronal and non-neuronal specific OCRs
Cell type
neuron
non−neuron
MDD
Control
Sum of neuronal and non-neuronal specific OCRs
K
Cell type
neuron
non−neuron
Diagnosis
MDD
Control
Principal component 2
Principal component 1
L
Sex
female
male
Diagnosis
MDD
Control
chrY read count
Fraction of reads in OCRs
M
Same person
yes
no
Density
Kinship score: called ATAC-seq genotype versus called ATAC-seq genotype
N
median percentile
46%
Density
OCR ranking (percentile)
O
Peak ID
OCR ranking
P
Randomized datasets
Original dataset
ROC
Sensitivity
Specificity
Nearest neighbour
Logistic regression
Naive Bayes
Random forest
SVM (linear kernel)
SVM (polynomial kernel)
Q
Promoter OCR -vs- Gene
(log10 number of combinations)
Pearson correlation coefficient
all
P−value < 0.05
FDR < 0.05
P−value range
R
PDE4B
PP1R16B
CREB5
NCOR2
PMFBP1
HLA-DBQ1
ARHGEF3
SUSD1
Normalized Fold Change
Control
MDD

Supplemental Figure 1. Quality control metrics for human postmortem MDD molecular profiling, related to Figure 1

(**A**) Principal component analysis of sample gene expression levels. (**B**) Analysis of scale-free fit index for possible soft-thresholding powers (β). (**C**) Analysis of mean connectivity for possible soft-thresholding powers. (**D**) GO analysis for 1,450 DE genes between MDD and control groups, separated by up/down regulation. (**E**) Mean insert size distribution. For all whisker plots in this figure: The center line indicates the median, the box shows the interquartile range, whiskers indicate the highest/lowest values within 1.5x the interquartile range. (**F**) Distance-to-TSS distribution of OCRs. (**G**) Fragment length distribution. (**H**) Correlations of raw reads coverage (number of reads) over consecutive bins of 10,000 bp genomic regions between samples originating from the same person versus a different person. (**I**) TSS enrichment. (**J**) Enrichment of cell-type specific signals in ATAC-seq samples calculated as a difference between the number of normalized reads within the ten most significant neuronal and the ten most significant non-neuronal specific OCRs from a previous analysis of prefrontal cortex-derived samples. (**K**) Principal component analysis of sample expression levels in OCRs. (**L**) Sex check showing the number of reads mapped chromosome Y as a function of the number of all reads in OCRs. (**M**) Genotype check based on pair-wise comparison of genotypes called from ATAC-seq samples. Pairs of neuronal and non-neuronal samples supposedly originating from the same person have distinctly higher scores (green line) than pairs of samples from different individuals (yellow line). (**N**) Summary and (**O**) per-OCR distribution of *P*-value ranking for the reported set of 203 differentially accessible OCRs within differentially analyses results generated on the datasets of non-neuronal samples with randomly permuted MDD and Control status (n=100 permuted datasets). This analysis proves that the reported set of 203 differentially accessible OCRs (median percentile of *P*-value is 1%) is not affected by technical artifacts since their median percentile of *P*-value in the datasets with permuted MDD and Control status is 46% (further details in Methods: Differential analysis of chromatin accessibility). (**P**) Performance of machine learning classifiers built on the reported set of 203 differential OCRs and 203 random OCRs. To enable the robust performance evaluation, the repeated 5-fold cross-validation was applied ($k_{repeat} = 10$); additionally, the whole process was repeated 10 times with different sets of 203 randomly selected OCRs. (**Q**) Correlation between t-statistics of MDD-associated changes in the epigenome (ATAC-seq, where the given OCR overlaps the gene's TSS) and MDD-associated changes in transcription (RNA-seq). The *P*-value threshold of filtering ATAC-seq OCRs is shown on the x-axis (*P*-value calculated by two-sided t-test), and the Pearson correlation coefficient is shown on the y-axis. The size of the point denotes the log10 count of promoter-gene combinations. (**R**) Normalized fold change of mRNA expression in FANS-sorted non-neuronal nuclei from MDD vs.

control OFC tissue for the listed gene targets of MDD-specific OCRs identified in the ATAC-seq dataset. For all whisker plots in this figure: The center line indicates the median, the box shows the interquartile range, whiskers indicate the highest/lowest values within 1.5x the interquartile range. Student's two-tailed t-tests were performed for statistical comparisons, *=p<.05, **=p<.01. Data displayed as mean (+/- SEM).

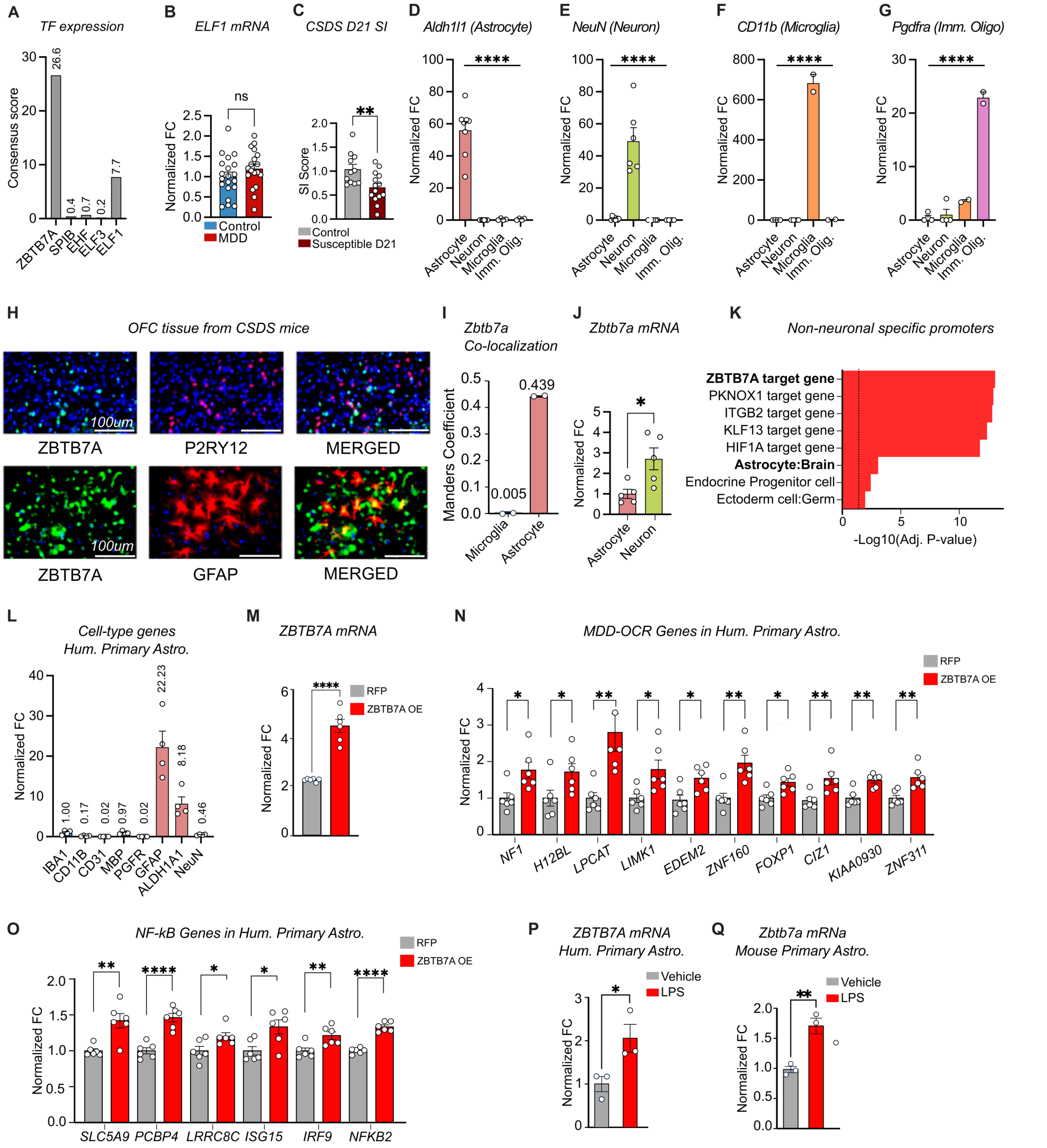

Supplemental Figure 2. Identification and validation of a ZBTB7A as a key transcription factor regulating MDD-specific OCRs, related to Figure 2

(**A**) Consensus score from the Human Protein Atlas[1] for expression in human brain for each factor. The mRNA expression data is derived from deep sequencing of RNA (RNA-seq) from 37 different normal tissue types. (**B**) Normalized fold change for mRNA expression for ELF1 in bulk human

OFC tissues, control *vs.* MDD. (**C**) Social interaction ratio for control (n=11) vs. stress susceptible (n=14) CSDS mouse groups at 21 d post-stress. (**D**) qPCR expression data for astrocyte-specific gene *Aldh1a1* in MACs-isolated cell fractions (**E**) qPCR expression data for neuron-specific *Rbfox3* (*NeuN* in MACs-isolated cell fractions). (**F**) qPCR expression data for *Cd11b* in MACs-isolated microglia fractions. (**G**) qPCR expression data for *Pdgfra* in MACs-isolated immature oligodendrocyte fractions. (**H**) 20x IHC images showing Zbtb7a protein is expressed in mouse OFC astrocytes, depicts overlap of Zbtb7a with astrocyte-specific marker Gfap. (**I**) Thresholded Mander's coefficient describes overlap of color channels of interest. (**J**) qPCR expression data for *Zbtb7a* in MACs-isolated astrocyte vs. neuron cell fractions. (**K**) GO analysis with CellMarker Augmented Database and CHEA ENCODE Consensus database for genes in detected non-neuronal specific promoters, filtered by logFC > 1, (+/-) 3000bp from TSS. (**L**) Normalized fold change of cell-type specific marker genes in human primary astrocyte-enriched cultures. (**M**) Expression of *ZBTB7A* mRNA in human primary cultured astrocytes treated with *ZBTB7A* OE lentivirus *vs.* RFP empty vector control virus. (**N-O**) Bar graph showing normalized fold change of mRNA expression in ZBT-OE vs. RFP human primary cultured astrocytes for the listed gene targets. (**P**) Normalized fold change of *ZBTB7A* mRNA expression in cultured human astrocytes treated with saline vs. LPS. (**Q**) Normalized fold change of *Zbtb7a* mRNA expression in cultured mouse astrocytes treated with saline vs. LPS. Student's two-tailed t-tests or 1-way ANOVA with MC tests were performed for statistical comparisons. Data presented as mean (+/- SEM). *=p<.05, **=p<.01, ***=p<.001, ****=p<.0001.

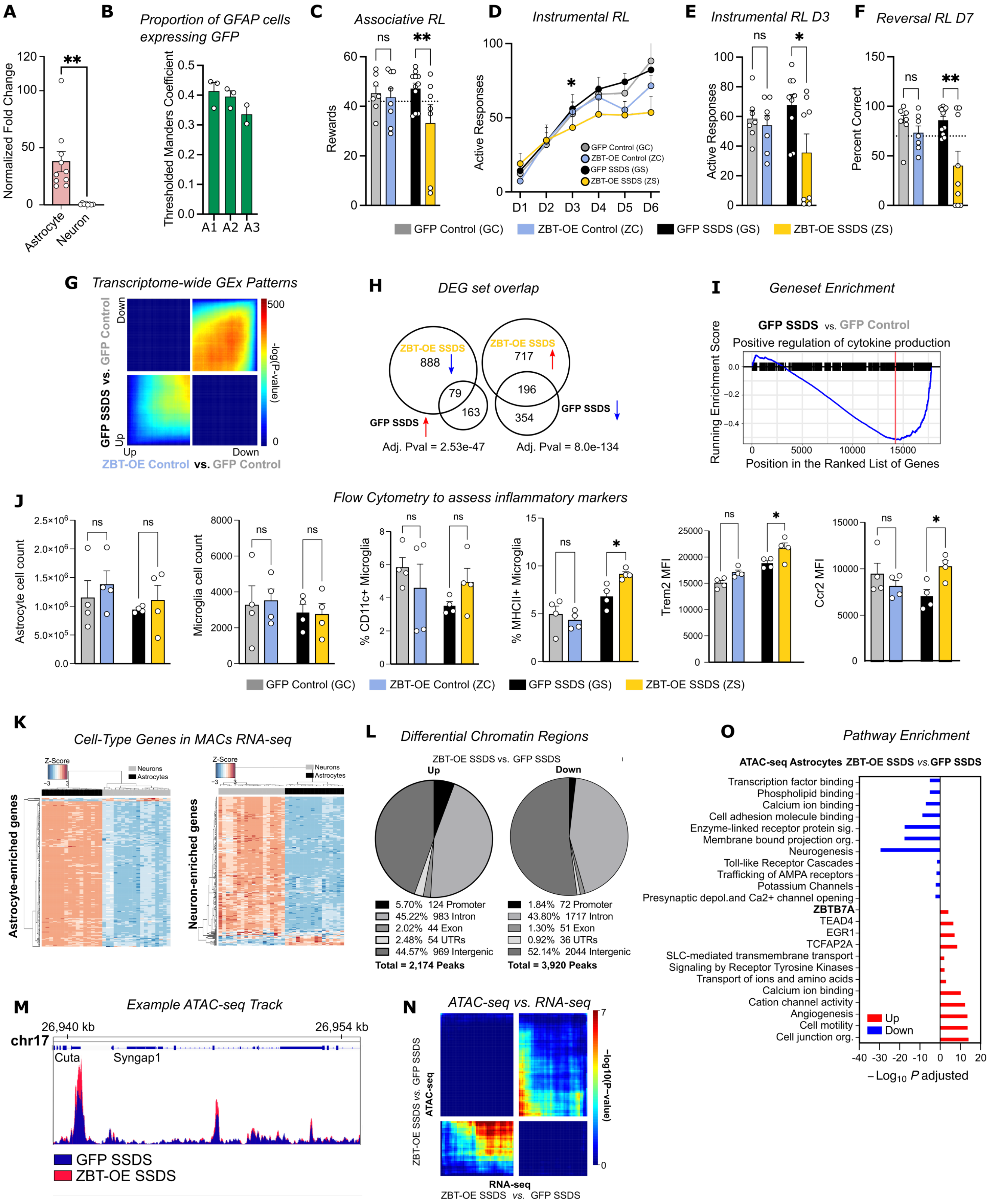
A
Normalized Fold Change
**
Astrocyte
Neuron
B
Proportion of GFAP cells expressing GFP
Thresholded Manders Coefficient
A1 A2 A3
C
Associative RL
Rewards
ns
**
D
Instrumental RL
Active Responses
*
GFP Control (GC)
ZBT-OE Control (ZC)
GFP SSDS (GS)
ZBT-OE SSDS (ZS)
D1 D2 D3 D4 D5 D6
E
Instrumental RL D3
Active Responses
ns
*
F
Reversal RL D7
Percent Correct
ns
**
GFP Control (GC)
ZBT-OE Control (ZC)
GFP SSDS (GS)
ZBT-OE SSDS (ZS)
G
Transcriptome-wide GEx Patterns
GFP SSDS vs. GFP Control
ZBT-OE Control vs. GFP Control
Up
Down
-log(P-value)
H
DEG set overlap
ZBT-OE SSDS
888
79
163
GFP SSDS
ZBT-OE SSDS
717
196
354
GFP SSDS
Adj. Pval = 2.53e-47
Adj. Pval = 8.0e-134
I
Geneset Enrichment
GFP SSDS vs. GFP Control
Positive regulation of cytokine production
Running Enrichment Score
Position in the Ranked List of Genes
J
Flow Cytometry to assess inflammatory markers
Astrocyte cell count
Microglia cell count
% CD11c+ Microglia
% MHCII+ Microglia
Trem2 MFI
Ccr2 MFI
ns
*
GFP Control (GC)
ZBT-OE Control (ZC)
GFP SSDS (GS)
ZBT-OE SSDS (ZS)
K
Cell-Type Genes in MACs RNA-seq
Z-Score
Neurons
Astrocytes
Astrocyte-enriched genes
Neuron-enriched genes
L
Differential Chromatin Regions
ZBT-OE SSDS vs. GFP SSDS
Up
Down
5.70% 124 Promoter
45.22% 983 Intron
2.02% 44 Exon
2.48% 54 UTRs
44.57% 969 Intergenic
Total = 2,174 Peaks
1.84% 72 Promoter
43.80% 1717 Intron
1.30% 51 Exon
0.92% 36 UTRs
52.14% 2044 Intergenic
Total = 3,920 Peaks
O
Pathway Enrichment
ATAC-seq Astrocytes ZBT-OE SSDS vs. GFP SSDS
Transcription factor binding
Phospholipid binding
Calcium ion binding
Cell adhesion molecule binding
Enzyme-linked receptor protein sig.
Membrane bound projection org.
Neurogenesis
Toll-like Receptor Cascades
Trafficking of AMPA receptors
Potassium Channels
Presynaptic depol.and Ca2+ channel opening
ZBTB7A
TEAD4
EGR1
TCFAP2A
SLC-mediated transmembrane transport
Signaling by Receptor Tyrosine Kinases
Transport of ions and amino acids
Calcium ion binding
Cation channel activity
Angiogenesis
Cell motility
Cell junction org.
Up
Down
−Log10 P adjusted
M
Example ATAC-seq Track
26,940 kb
26,954 kb
chr17
Cuta
Syngap1
GFP SSDS
ZBT-OE SSDS
N
ATAC-seq vs. RNA-seq
ZBT-OE SSDS vs. GFP SSDS
ATAC-seq
RNA-seq
ZBT-OE SSDS vs. GFP SSDS
-log10(P-value)

Supplementary Figure 3. ZBTB7A OE in OFC astrocytes causes significant changes in behavior, chromatin accessibility and gene expression, related to Figure 3

(**A**) Normalized fold change of qPCR GFP transgene expression in MACs-isolated astrocytes and neurons from OFC tissues transduced with ZBTB7A OE virus *vs.* GFP, n = 10/group. (**B**) Proportion of GFAP cells expressing GFP in OFC Layer 5 transduced with AAV6-GFAP-GFP virus quantified via Mander's Co-localization coefficient, per animal "A" = individual mouse. (**C**) Individual values for Day 2 of task shown in (3H). 2-way ANOVA, interaction ($p = 0.0140$). Sidak's MC test, GFP control vs. GFP SSDS (ns, $p=0.8452$). GFP SSDS vs. ZBT-OE SSDS ($p = .0059$). ZBT-OE control vs. ZBT-OE SSDS ($p = 0.0116$), GFP control vs. GFP SSDS ($p=0.761$). Dotted line shows criterion value (~60%). (**D**) Instrumental reward learning (RL) task. "D" indicates Day of test. 3-way ANOVA, main effect of Test Day x Virus [$p = 0.0182$]. (**E**) Individual values for day 3 of task shown in (D). 2-way ANOVA, main effect of virus [$p= 0.0453$]. (**F**) Individual values for Day 7 as shown in (Figure 3I). 2-way ANOVA, main effect of virus [$p = 0.0054$]. (**G**) RRHO comparing gene expression overlaps between indicated control comparisons. (**H**) Venn-diagram and odds ratio test of the overlap between DEGs in bulk OFC tissues comparing ZBT-OE stress vs. GFP SSDS, with GFP SSDS vs. GFP control. Note for GFP SSDS vs. GFP control, DEGs were defined at pval < 0.05 (**I**) GSEA enrichment plot for most significantly enriched gene set in GFP SSDS vs. GFP Control in bulk OFC tissue. (**J**) Number of astrocytes [left], and microglia per organ. Percent CD11c+ microglia [far left], percent MHCII+ microglia [left], Trem2 MFI and Ccr2 [far right] MFI in virally transduced ZBT-OE vs. GFP mice (+/- SSDS) OFC via flow cytometry, n = 4/group. Gating strategy shown in **Supplementary Fig. 6.** (**K**) Heatmaps depict unsupervised clustering of normalized read count values in MACs-isolated astrocytes and neurons for (left) 239 astrocyte-enriched genes and (right) 279 neuron enriched genes identified in previous report[5]. (**L**) ATAC-seq diffReps analysis of differential accessibility comparing ZBT-OE SSDS vs. GFP SSDS. Pie charts indicate distribution of differential accessibility events, stratified by genomic context. (**M**) Representative pile-up traces of cell specific ATAC-seq signal overlapping Syngap1 gene. (**N**) RRHO comparing gene expression overlaps of MACs-isolated astrocytes with MACS-isolated astrocyte chromatin accessibility for indicated conditions. (**O**) GO pathway analysis of gene targets associated with differentially expressed [red is more accessible, blue is less accessible] chromatin regions between ZBT-OE SSDS and GFP OE SSDS. Data were analyzed with Student's two-tailed t-tests or with 2-way ANOVA, or 3-way ANOVA, followed by 2-Way ANOVAs for MC comparisons, *=$p<.05$, **=$p<.01$. All data graphed as means ± SEM.

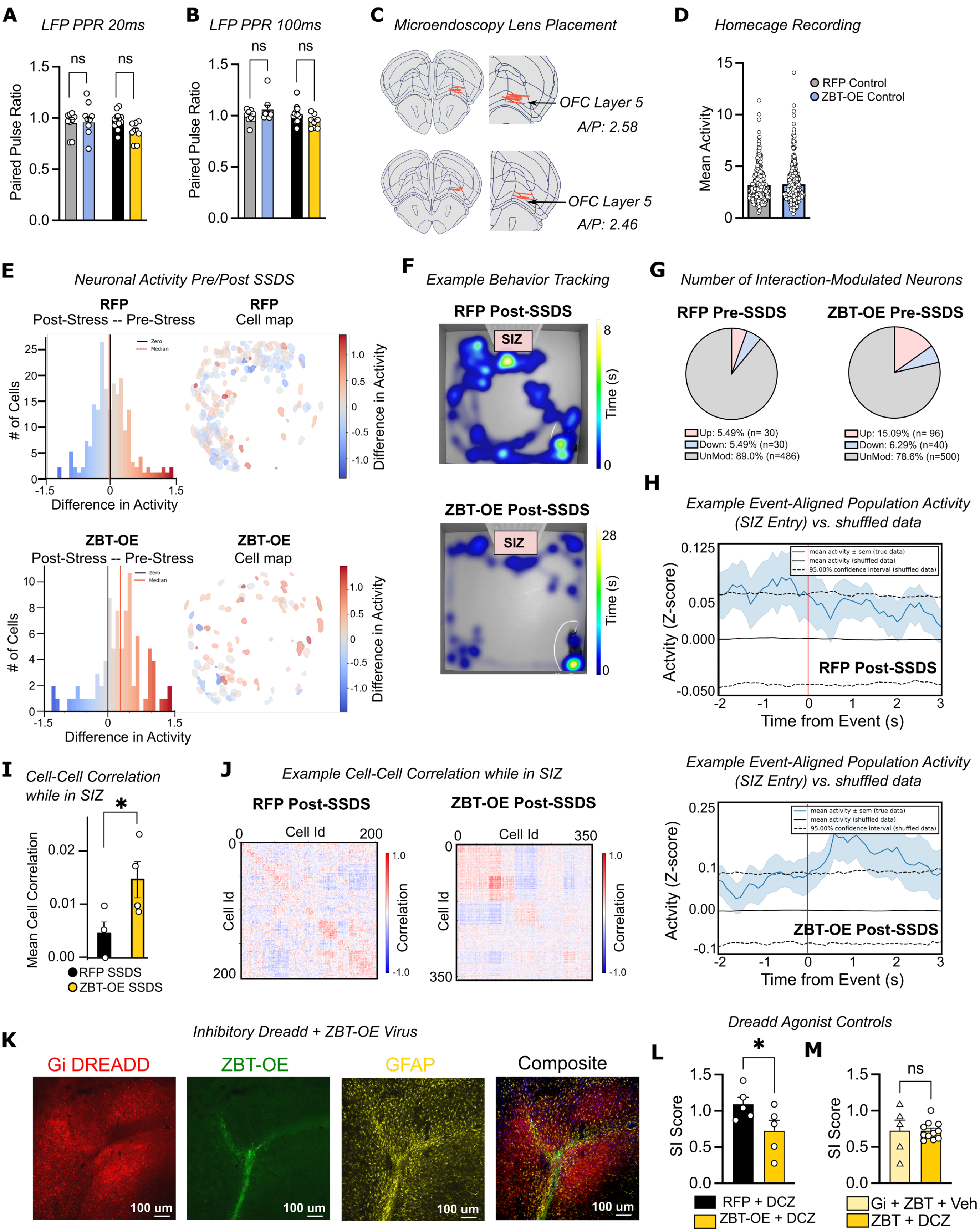

A
LFP PPR 20ms
Paired Pulse Ratio
ns
ns
B
LFP PPR 100ms
C
Microendoscopy Lens Placement
OFC Layer 5
A/P: 2.58
OFC Layer 5
A/P: 2.46
D
Homecage Recording
Mean Activity
RFP Control
ZBT-OE Control
E
Neuronal Activity Pre/Post SSDS
RFP
Post-Stress -- Pre-Stress
RFP
Cell map
# of Cells
Difference in Activity
ZBT-OE
Post-Stress -- Pre-Stress
ZBT-OE
Cell map
F
Example Behavior Tracking
RFP Post-SSDS
SIZ
Time (s)
ZBT-OE Post-SSDS
G
Number of Interaction-Modulated Neurons
RFP Pre-SSDS
ZBT-OE Pre-SSDS
Up: 5.49% (n= 30)
Down: 5.49% (n=30)
UnMod: 89.0% (n=486)
Up: 15.09% (n= 96)
Down: 6.29% (n=40)
UnMod: 78.6% (n=500)
H
Example Event-Aligned Population Activity
(SIZ Entry) vs. shuffled data
mean activity ± sem (true data)
mean activity (shuffled data)
95.00% confidence interval (shuffled data)
Activity (Z-score)
RFP Post-SSDS
Time from Event (s)
Example Event-Aligned Population Activity
(SIZ Entry) vs. shuffled data
ZBT-OE Post-SSDS
I
Cell-Cell Correlation
while in SIZ
*
Mean Cell Correlation
RFP SSDS
ZBT-OE SSDS
J
Example Cell-Cell Correlation while in SIZ
RFP Post-SSDS
ZBT-OE Post-SSDS
Cell Id
Correlation
K
Inhibitory Dreadd + ZBT-OE Virus
Gi DREADD
ZBT-OE
GFAP
Composite
100 um
Dreadd Agonist Controls
L
*
SI Score
RFP + DCZ
ZBT-OE + DCZ
M
ns
Gi + ZBT + Veh
ZBT + DCZ

Supplemental Figure 4. Population electrophysiology, cellular resolution calcium imaging, and chemogenetic manipulation in the context of astrocyte-specific ZBT OE and SSDS, related to Figure 4

(**A**) Individual values for Local Field Potential (LFP) Paired-Pulse Ratio (PPR) at 20ms. (**B**) Individual values for Local Field Potential (LFP) Paired-Pulse Ratio (PPR) at 100ms. (**C**) Left: Histology summary of lens placements for all cellular resolution calcium imaging mice (all plotted on left hemisphere for visualization). Right: Zoom in on Layer 5 OFC for lens locations (N = 8 mice). (**D**) Mean Activity (Delta F/F) during baseline homecage recording. Values represent average neural activity over 5 minute period after 5 minutes habituation. (**E**) Example change in Neural Activity (Delta F/F) for the same individual neurons registered across social interaction recordings pre-SSDS and post-SSDS for one RFP mouse (top) and one ZBT-OE mouse (bottom). Left side histogram bins each cell in the cell map based on the pairwise difference in event rate, colored by relative change across two epochs (Post-SSDS – Pre-SSDS). The median is indicated by red line. Right: a cell map of the cells in the population, with the cells colored by magnitude of relative change, with a colorbar mapping the relative change. (**F**) Example behavior tracking on videos time locked to the calcium imaging recordings in order to identify neurons that are significantly modulated by entry into the Social interaction zone. (**G**) Proportion and number of neurons significantly modulated by aggressor proximity through entry into the social interaction zone Pre-SSDS. (**H**) Example for one RFP and one ZBT-OE mouse showing the average z-scored neural activity for all neurons in the cell map (blue trace) around the SI entry event (red vertical line). The blue line represents the mean signal and the shaded light blue region represents the standard error of mean (SEM). The two black lines represent the upper and lower of the 95% confidence interval of the null distribution obtained from the circular permutation test using shuffling.(**I**) Comparison of the fraction of neurons in each modulation category between the two viral groups Post-SSDS (p=0.0361). (**J**) Example heat maps for pairwise Pearson correlation between each cell in the cell set for one RFP animal (top) and one ZBT-OE animal (bottom). Heatmaps are organized using hierarchical clustering so that groups of highly correlated variables are next to each other. The diagonal entries in the correlation matrices are set to zero. (**K**) IHC validation of hsyn-hM4D(Gi)-mCherry (in red) and GFAP-ZBT OE (in green) localized in astrocytes (GFAP, in yellow) and DAPI (in blue). Images taken at 10x magnification. (**L**) Social interaction score for GFP vs. ZBT-OE SSDS groups, both injected with DCZ to demonstrate that the DREADD agonist on its own does not affect previously observed phenotypes. (**M**) Social interaction score for ZBT-OE SSDS + DCZ vs. ZBT-OE + G_i Dreadd + vehicle, demonstrating that Gi Dreadd expression on it's own with no agonist injection does not affect previously observed

phenotypes. Data were analyzed with Student's two-tailed t-tests or with 1-way ANOVA plus Tukey's MC test, *=p<.05, **=p<.01, ***=p<.001, ****=p<.0001. All data graphed as means ± SEM.

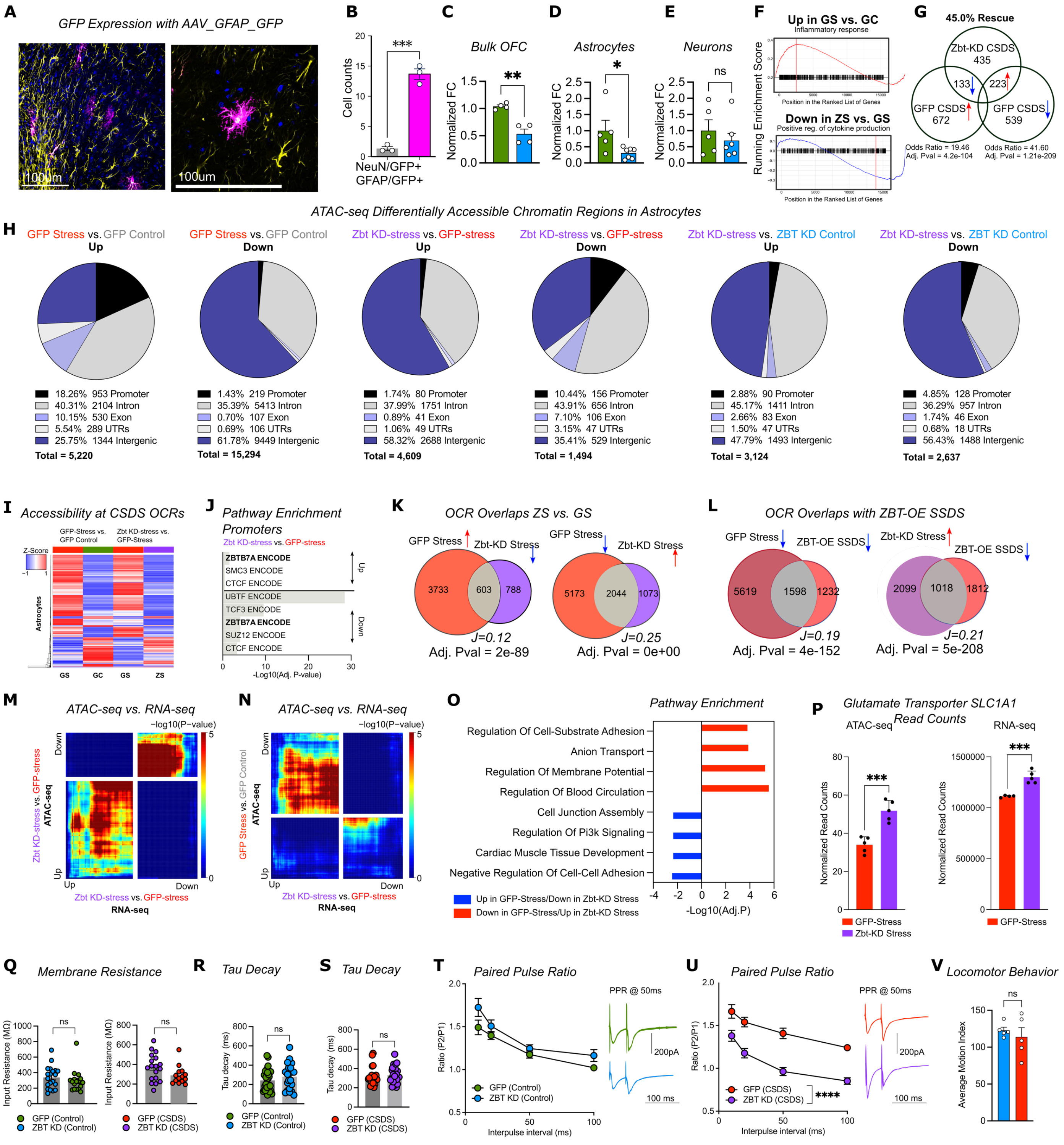

Supplemental Figure 5. Zbtb7a KD alters cell-type specific chromatin accessibility and gene expression, related to Figure 5

(**A**) Representative IHC images of OFC tissues transduced with an rAAV6 virus expressing ZBTB7A-GFP (in magenta) overlaid with a nuclear co-stain (DAPI in blue) and GFAP (in yellow) to show astrocyte-specific expression. (**B**) Number of cells in OFC tissues transduced with AAV6-ZBTB7A-GFP that are co-expressing GFAP/GFP or NEUN/GFP. (**C**) Normalized fold change of qPCR *Zbtb7a* gene expression from OFC tissues transduced with Zbt-KD virus vs. miR-neg-GFP (GFP), with n = 4/group. (**C**) qPCR expression levels of the Zbtb7a mRNA in bulk OFC tissue transduced with AAV6-GFAP-miR-KD. (**C-D**) qPCR expression levels of the Zbtb7a mRNA in MACs-isolated (**C**) astrocytes and (**D**) neurons from OFC tissue transduced with AAV6-GFAP-miR-Zbt-KD. (**E**) GSEA enrichment plot for most significantly enriched gene set in GFP CSDS vs. GFP Control and ZBT CSDS vs. GFP CSDS in bulk OFC tissue. (**G**) Venn-diagram and odds ratio test of the overlap between DE genes in bulk OFC RNA-seq comparing Zbt-KD CSDS vs. GFP CSDS, with GFP CSDS vs. GFP control. (**H**) ATAC-seq diffReps analysis of differential accessibility between indicated conditions. Pie charts indicate distribution of differential accessibility events, stratified by genomic context for the indicated conditions and separated for up/down events. (**I**) Clustering of groups at 1,138 overlapping genomic regions between GFP CSDS vs. GFP control and Zbt-KD CSDS vs. GFP CSDS, depicting Z-score of log2FC accessibility (**J**) Gene ontology (GO) pathway analysis of differentially accessible promoters from Zbt-KD CSDS vs. GFP CSDS [less accessible promoters, top] and GFP CSDS vs. GFP control [more accessible promoters, bottom]. (**K**) Venn diagram and odds ratio analyses of the number of shared and distinct OCR gene targets between indicated conditions depicts Zbt-KD rescue of GFP-CSDS OCR gene targets. (**L**) Venn diagram and odds ratio analyses of the number of shared and distinct OCR gene targets between indicated conditions depicts shared differentially accessible peaks between GFP CSDS and ZBT-OE SSDS, and Zbt-KD rescue of ZBT-OE peaks. Numbers indicate differentially accessible peaks, "*J*" indicates the Jaccard index. (**M-N**) RRHO comparing gene expression and chromatin accessibility for the indicated comparisons. (**O**) GO pathway analysis of rescued OCR gene targets between Zbt-KD CSDS and GFP CSDS MACS-isolated astrocytes ATAC-seq. (**P**) Normalized read counts for accessibility (left) and gene expression (right) at the Slc1a2 gene in MACS-isolated astrocytes. (**Q**) Membrane Resistance. (**R-S**) EPSP rate of decay (Tau). (**T-U**) Paired-Pulse Ratio. (**V**) Average motion index during a locomotor control test, measured by video freeze software inside the same behavioral apparatus used to perform reward-learning tasks. Data were analyzed with Student's two-tailed t-tests. *=p<.05, **=p<.01, ***=p<.001, ****=p<.0001. "J" indicates Jacquard Index. All data graphed as means ± SEM.

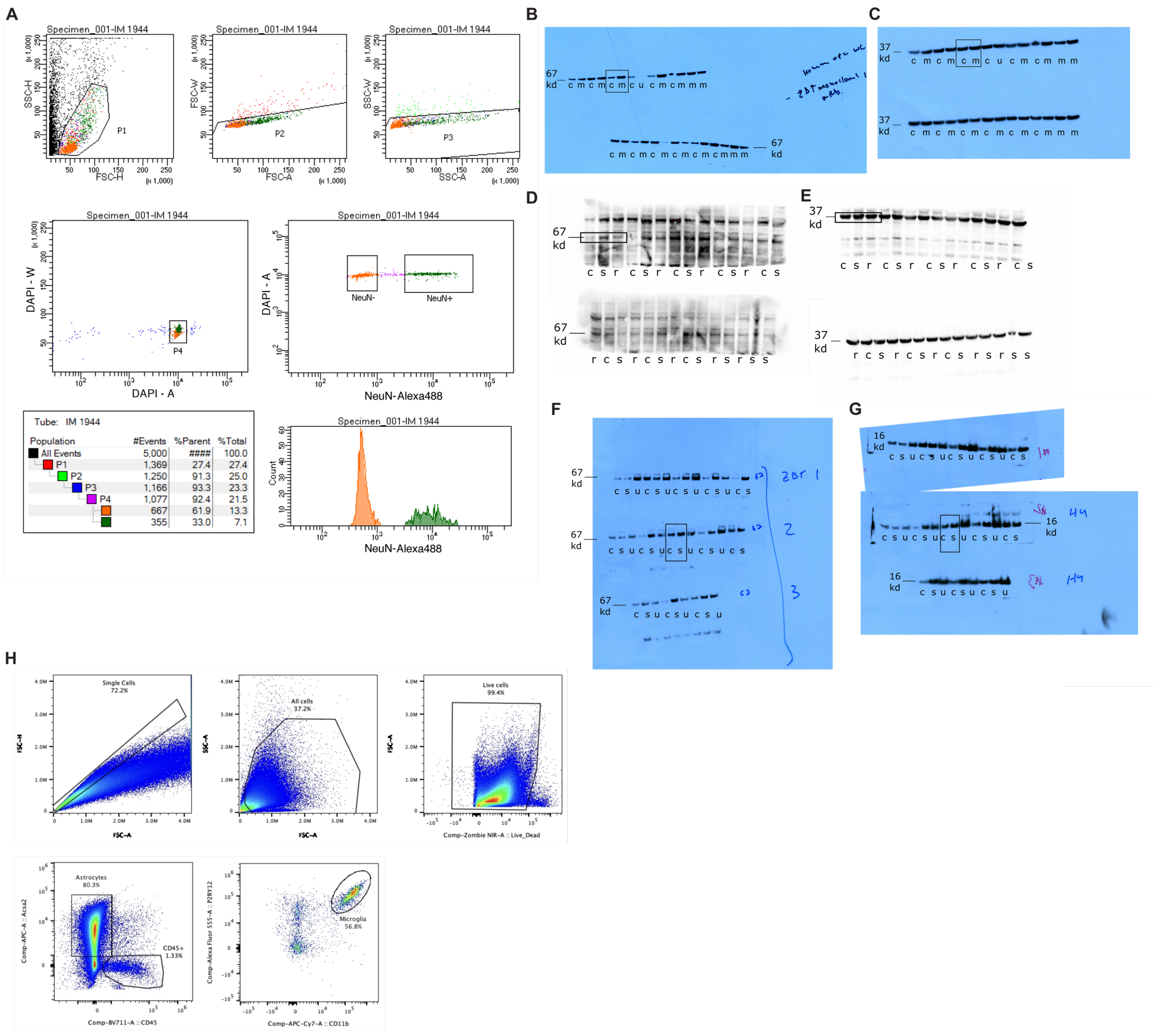

Supplemental Figure 6. Flow cytometry gating and raw blots, related to STAR methods

(**A**) For FANS-coupled ATAC-seq on human postmortem tissues, nuclear populations were initially gated by side and forward scatter to differentiate nuclei from cellular debris. Populations were then gated based on DAPI staining to identify singlets and to further disregard debris. DAPI positive nuclei were subsequently gated based on NeuN staining to differentiate neurons (NeuN$^+$) from non-neurons (NeuN$^-$). Final nuclei population abundance for non-Neurons (NeuN$^-$): 70.5% (in orange) and for neurons (NeuN$^+$): 29.5% (in green). (**B**) Western blot film scan for ZBTB7A in

bulk human OFC tissue, MDD (labeled "m") vs. controls (labeled "c"). ZBTB7A band at expected molecular weight of 67kDa. Note samples labeled "u" are not included in this manuscript due to lack of signal (suspected improper nuclear lysis). (**C**) Western blot film scan for housekeeping gene GAPDH in human OFC, MDD vs. controls. Run on the same membrane as ZBTB7A in (B). (**D**) Raw image from chemidoc for western blot film for Zbtb7a in male mouse OFC, 48 hours after final defeat. CSDS susceptible (labeled "s") vs. CSDS resilient (labeled "r") vs. controls (labeled "c"). (**E**) Raw image from chemidoc western blot film for Gapdh loading control in male mouse OFC, CSDS susceptible vs. resilient vs. controls. Run on the same membrane as Zbtb7a in (D). (**F**) Western blot film scan for Zbtb7a in male mouse OFC, 21 days after final defeat. CSDS susceptible (labeled "s") vs. controls (labeled "c"). Note samples labeled "u" are from an unrelated study, and not included in this manuscript. (**G**) Western blot film scan for H3.3 loading control in male mouse OFC, CSDS susceptible *vs.* controls (note H3.3 was used for these blots due to use of nuclear lysates, Gapdh could not be used). Run on the same membrane as Zbtb7a in (F). (**H**) Gating strategy used to identify cell populations in the OFC of mouse OE experiments (Fig. S4).
